# Fast evaluation of protein dynamics from deficient ^15^N relaxation data

**DOI:** 10.1007/s10858-018-0176-3

**Published:** 2018-03-28

**Authors:** Łukasz Jaremko, Mariusz Jaremko, Andrzej Ejchart, Michał Nowakowski

**Affiliations:** 1 Division of Biological and Environmental Sciences and Engineering, King Abdullah University of Science and Technology (KAUST), Thuwal, 23955-6900 Kingdom of Saudi Arabia; 2 Institute of Biochemistry and Biophysics, Polish Academy of Science, Pawinskiego 5A, 02-106 Warszawa, Poland; 3 Faculty of Chemistry, Biological and Chemical Research Centre, University of Warsaw, Żwirki i Wigury 101, 02-089 Warszawa, Poland

**Keywords:** ^15^N magnetic relaxation, Protein dynamics, Model-free approach, Ratio, product, and difference of relaxation rates, Semi-quantitative analysis of ^15^N relaxation data

## Abstract

Simple and convenient method of protein dynamics evaluation from the insufficient experimental ^15^N relaxation data is presented basing on the ratios, products, and differences of longitudinal and transverse ^15^N relaxation rates obtained at a single magnetic field. Firstly, the proposed approach allows evaluating overall tumbling correlation time (nanosecond time scale). Next, local parameters of the model-free approach characterizing local mobility of backbone amide N–H vectors on two different time scales, *S*^2^ and *R*_*ex*_, can be elucidated. The generalized order parameter, *S*^2^, describes motions on the time scale faster than the overall tumbling correlation time (pico- to nanoseconds), while the chemical exchange term, *R*_*ex*_, identifies processes slower than the overall tumbling correlation time (micro- to milliseconds). Advantages and disadvantages of different methods of data handling are thoroughly discussed.

**Electronic supplementary material:**

The online version of this article (10.1007/s10858-018-0176-3) contains supplementary material, which is available to authorized users.

## Introduction

Analysis of nuclear magnetic relaxation provides insight into the molecular motions of proteins (Palmer 2004; Mayo and Daragan 2005; Ejchart 2007; Charlier et al. 2016; Lisi and Loria 2016). One of the most successful and widely used approaches to the analysis of relaxation data is the model-free approach, MFA (Lipari and Szabo 1982). Two other model-independent descriptions of the internal molecular motions have been developed independently: the two-step model (Halle and Wennerström 1981) and the slowly relaxing local structure model (Tugarinov et al. 2001). In the frame of the MFA, local mobility is described by two parameters, the generalized order parameter, *S*^2^, which corresponds to the spatial freedom of motion, and the internal correlation time, *τ*_int_, corresponding to the rate of this motion in the pico- to nanosecond time scale, which is smaller than the single correlation time describing an isotropic overall molecular tumbling, *τ*_*R*_. The additional term, *R*_*ex*_, accounts for the conformational exchange contribution to *R*_2_ resulting from processes in the micro- to millisecond time scale, often referred to as chemical exchange effects (Stone et al. 1992; Korzhnev et al. 2001).

The most frequently measured protein relaxation parameters are the longitudinal, *R*_1_, and transverse, *R*_2_, relaxation rates of backbone amide ^15^N nuclei complemented with ^15^N-{^1^H} *NOE*s (Palmer 2004; Reddy and Rainey 2010). Nuclear magnetic relaxation measurements are long lasting, their data reduction is demanding, and interpretation of the results complex (Zhukov and Ejchart 1999; Pawley et al. 2006; Jaremko et al. 2015). Especially *NOE* measurements are very time consuming owing to their inherently low sensitivity (Fushman 2003) and susceptibility to systematic errors resulting from not fully relaxed spectra and/or saturation transfer due to the exchange with water protons (Ferrage et al. 2008; Gong and Ishima 2007; Grzesiek and Bax 1993; Renner et al. 2002). So it often happens that limited relaxation data (e.g., *R*_1_ and *R*_2_ at a single magnetic field), precluding the determination of all MFA parameters, are solely available. Then, commonly used procedure is to examine the ratio between transverse and longitudinal rates, *R*_2_/*R*_1_, for the estimation of global isotropic correlation time of a protein (Kay et al. 1989). As a prerequisite this procedure requires an efficient rejection from calculation the residues exhibiting skewed *R*_2_/*R*_1_ values owing to intense fast local motions and/or slow conformational exchange. Residues with an *NOE* (if available) < 0.6 are usually excluded, since in this case the internal motions are likely to skew significantly the *R*_2_/*R*_1_ value. Usually such residues exhibit low experimental *R*_2_/*R*_1_ values. On the other hand, residues undergoing conformational exchange are characterized by high *R*_2_/*R*_1_ values. Routine method of appropriate residue selection can be done by excluding those residues with a *R*_2_/*R*_1_ value outside of ± 1 standard deviation of the mean (Clore et al. 1990).

With the *τ*_*R*_ already estimated, three methods of further analysis of limited relaxation data were independently developed. One of them is based on the observation that one of the MFA parameters, the generalized order parameter, *S*^2^, can be extracted with a reasonable accuracy from a linear combination of relaxation rates, 2*R*_2_ − *R*_1_ (Habazettl and Wagner 1995). Another method investigates the product of relaxation rates *R*_1_*R*_2_ also giving access to the *S*^2^ parameter (Kneller et al. 2002). The last method of the *S*^2^ parameter extraction from *R*_1_ and *R*_2_, measured with the CEST technique to save experimental time and named lean MFA (LMFA), relies on the least squares minimization (Gu et al. 2016). Since all these methods base on the determination of the overall correlation time *τ*_*R*_ from the *R*_2_/*R*_1_ ratio an appropriate data selection seems to be crucial. Therefore, it was proposed to use for this purpose the product of relaxation rates *R*_1_*R*_2_ and claimed that in contrary to the *R*_2_/*R*_1_ ratio, the former allows to distinguish between the effects of motional anisotropy and chemical exchange (Kneller et al. 2002). For convenience, *R*_2_/*R*_1_, 2*R*_2_ − *R*_1_, and *R*_1_*R*_2_ will be further denoted as *Q, D*, and *P*, respectively.

This paper describes a comparative analysis of three mentioned above combinations of relaxation rates, which allows to select the optimal method of the MFA parameter elucidation from deficient relaxation data. The utility of this approach is presented by applying it to the relaxation data of four proteins of different size: immunoglobulin-binding domain of streptococcal protein G, GB1 (Idiyatullin et al. 2003), human ubiquitin (Lee and Wand 1999), human S100A1 calcium binding protein in *apo* state (Nowakowski et al. 2011), and β-lactamase PSE-4 (Morin and Gagné 2009). First limited use of this approach was applied to the ^15^N relaxation rates of BacSp222 peptide (Nowakowski et al. 2018).

## Method

Equations describing relaxation rates of ^15^N nuclei relaxing by dipolar and chemical shift anisotropy mechanisms in terms of spectral density functions are given as (Abragam 1989; Korzhnev et al. 2001):

1 $${R_1}=\frac{1}{4}{d^2}\left[ {J\left( {{\omega _H} - {\omega _N}} \right)+3J\left( {{\omega _N}} \right)+6J\left( {{\omega _H}+{\omega _N}} \right)} \right]+\frac{1}{3}{c^2}J\left( {{\omega _N}} \right)$$

2 $${R_2}=\frac{1}{8}{d^2}\left[ {4J\left( 0 \right)+J\left( {{\omega _H} - {\omega _N}} \right)+3J\left( {{\omega _N}} \right)+6J\left( {{\omega _H}} \right)+6J\left( {{\omega _H}+{\omega _N}} \right)} \right]+\frac{1}{{18}}{c^2}\left[ {4J(0)+3J({\omega _N})} \right]+{R_{ex}}$$

where $d=\frac{{{\mu _0}}}{{8{\pi ^2}}}\frac{{{\gamma _N}{\gamma _H}h}}{{\left\langle {r_{{NH}}^{3}} \right\rangle }},\quad c={\omega _N}\Delta \sigma$ and other symbols have their usual meaning. It has to be mentioned that in all calculations the vibrationally averaged N–H distance, *r*_NH_ = 1.04 Å (Ottiger and Bax 1998) and the chemical shift anisotropy of the ^15^N chemical shift tensor Δδ = − 170 ppm (Yao et al. 2010) were used.

The conformational exchange contribution to the transverse relaxation rate, *R*_*ex*_, is proportional to the square of the ^15^N Larmor frequency, *ω*_*N*_. This term can be written as ${R_{ex}}=\Phi \omega _{N}^{2}$ (Peng and Wagner 1995). The proportionality factor Φ represents the effectiveness of conformational exchange processes and is independent on magnetic field strength facilitating direct comparison of chemical exchange terms determined at different magnetic field strengths for different proteins.

Model-free approach spectral density function takes the form (Lipari and Szabo 1982):

3 $$J\left( \omega \right)=\frac{2}{5}\left[ {\frac{{{S^2}{\tau _R}}}{{1+{{\left( {\omega {\tau _R}} \right)}^2}}}+\frac{{\left( {1 - {S^2}} \right)\tau }}{{1+{{\left( {\omega \tau } \right)}^2}}}} \right]$$

where ${\tau ^{ - 1}}=\tau _{R}^{{ - 1}}+\tau _{{\operatorname{int} }}^{{ - 1}}$. Performing the complete MFA analysis of relaxation data for *N*-residue protein one has to determine 3*N* local parameters, *S*^2^, *τ*_int_, and *R*_*ex*_ for each residue. Additionally one global parameter *τ*_*R*_ or six parameters characterizing either isotropic or fully anisotropic overall tumbling, respectively, have to be determined. Extension of the spectral density function for isotropic motion (Eq. 3) to the anisotropic one, based on the formalism developed by Woessner (1962), was implemented to the protein relaxation studies (Tjandra et al. 1995; Baber et al. 2001). Allowing for the positive degree of freedom of a computational task it means that besides *R*_1_, *R*_2_, and ^15^N-{^1^H} *NOE* at a single magnetic field, at least one additional set of relaxation parameters has to be measured. It happens, however, that the number of available relaxation data is insufficient, due to sample instability or lack of experimental time and additional data processing has to be applied (Jaremko et al. 2014). Often only *R*_1_ and *R*_2_ relaxation rates at a single magnetic field are at one’s disposal.

**Fig. 1:**
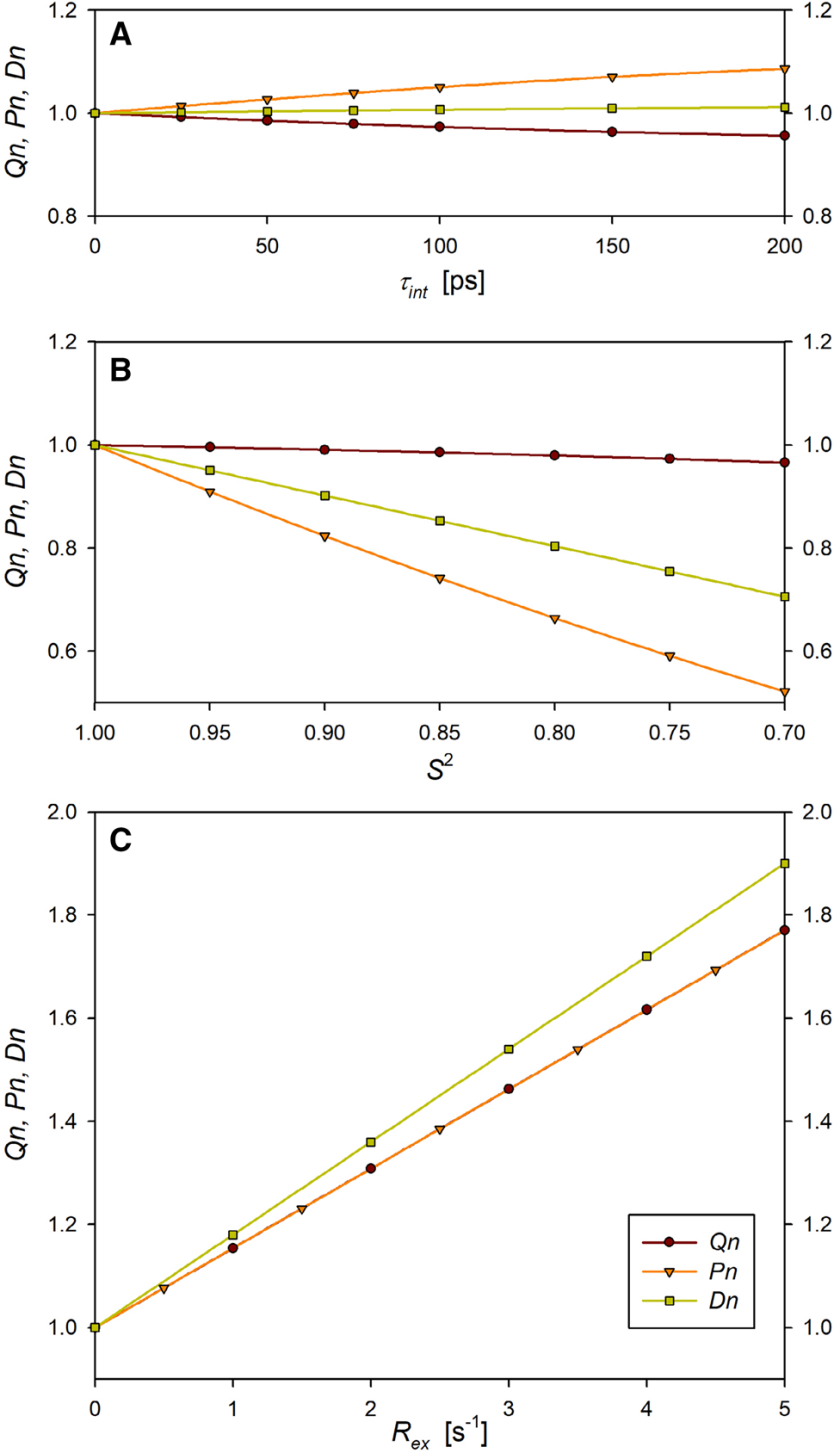
Calculated relationships between normalized *Q, D*, and *P* quantities and local parameters of MFA. The presented quantities are normalized in relation to their counterparts in rigid molecules. **A** *Q*(*τ*_int_), *D*(*τ*_int_), and *P*(*τ*_int_) functions with *S*^2^ = 0.85 and *R*_*ex*_ = 0. **B** *Q*(*S*^2^), *D*(*S*^2^), and *P*(*S*^2^) functions with *τ*_int_ = 50 ps and *R*_*ex*_ = 0. **C** *Q*(*R*_*ex*_), *D*(*R*_*ex*_), and *P*(*R*_*ex*_) with *τ*_int_ = 50 ps and *S*^2^ = 0.85. Additional input data: *τ*_R_ = 5 ns and B_0_ = 16.4 T were used in all calculations. Take note that *Q* and *P* are superposed in part **C**

A joined analysis of *Q* = *R*_2_*/R*_1_ and *D* = 2*R*_2_ − *R*_1_ or *P* = *R*_1_*R*_2_ values allows obtaining semi-quantitative insight into the protein dynamics owing to the different relations of these quantities to the MFA parameters and, therefore, untangling these parameters from experimental data. The *Q* parameter is quasi-insensitive to both local MFA parameters, *S*^2^ and *τ*_int_, in a reasonably broad range of their values (Fig. 1A, B). Therefore, it is well suited for the evaluation of the overall tumbling correlation times of proteins comprising residues with diverse local mobility. On the other hand, the *P* values are quasi-insensitive to *τ*_int_ but decrease considerably with the increased amplitude of local motions, as manifested at smaller values of the *S*^2^ order parameter (Fig. 1B). The *D* parameter is even less sensitive to *τ*_int_ changes than *Q* and *P*, but it displays a modest sensitivity to *S*^2^ changes. All three quantities, *Q, D*, and *P*, are sensitive to the chemical exchange term and increase with the *R*_*ex*_ enlargement (Fig. 1C). Simultaneous effect of *S*^2^ and *τ*_int_, changes on *Q, P* and *D* is shown in Figs. S1–S3 (Supporting Information). One has to be aware of the opposite effects of fast (ps–ns) and slow (µs–ms) motions on the *P* values. Both these effects can compensate one another leaving the *P* value unchanged and, thus, hiding chemical exchange effect. The *D* values are also sensitive to such compensation. They are, however, less sensitive to fast motions and more sensitive to slow ones than *P* values and, therefore, should retain at least partially the ability of detection of *R*_*ex*_ terms.

Use of *Q, D*, and *P* values in the analysis of a backbone protein dynamics requires several simplifying assumptions bearing a number of consequences. It has been noticed (Peng and Wagner 1992) that the spectral density functions at three highest frequencies *J*(*ω*_*H*_ + *ω*_*N*_), *J*(*ω*_*H*_), and *J*(*ω*_*H*_ − *ω*_*N*_) are only a small fraction of two other component *J*(0) and *J*(*ω*_*N*_) and can be neglected in Eqs. (1) and (2) describing *D* values (Habazettl and Wagner 1995) or *P* values (Kneller et al. 2002). As a result following expressions can be written:

4a $$Q=\frac{2}{3}\frac{{J(0)}}{{J({\omega _N})}}+\frac{1}{2}$$

4b $$D={A_1}J(0)$$

4c $$P={A_2}J(0)J({\omega _N})+{A_3}{[J({\omega _N})]^2}$$

$${\text{where }}{A_1}=\left( {{d^2}+\frac{4}{9}{c^2}} \right);{\text{ }}{A_2}=6{\left( {\frac{{{d^2}}}{4}+\frac{{{c^2}}}{9}} \right)^2};{\text{ }}{A_3}=\frac{1}{2}{\left( {\frac{3}{4}{d^2}+\frac{1}{3}{c^2}} \right)^2}$$

In the approach utilizing the *Q* ratio for the estimation of global correlation time, the assumption *τ*_int_ = 0.0 is made resulting in a simplified spectral density function (Kay et al. 1989).

5 $$J(\omega )=\frac{2}{5}\left[ {\frac{{{S^2}{\tau _R}}}{{1+{{(\omega {\tau _R})}^2}}}} \right]$$

In order to obtain so estimated global correlation time, *τ*_*R*_(*Q*), one has to compute it from Eq. (8) given by Kay et al. (1989). Additionally, neglecting second term in Eq. (4c) and assuming ${({\omega _N}{\tau _R})^2}>>1$ one obtains:

6a $$Q=\frac{2}{3}{\left( {{\omega _N}{\tau _R}} \right)^2}+\frac{1}{2}$$

6b $$D={A_1}{S^2}{\tau _R}$$

6c $$P={A_2}{\left( {\frac{{{S^2}}}{{{\omega _N}}}} \right)^2}$$

**Fig. 2:**
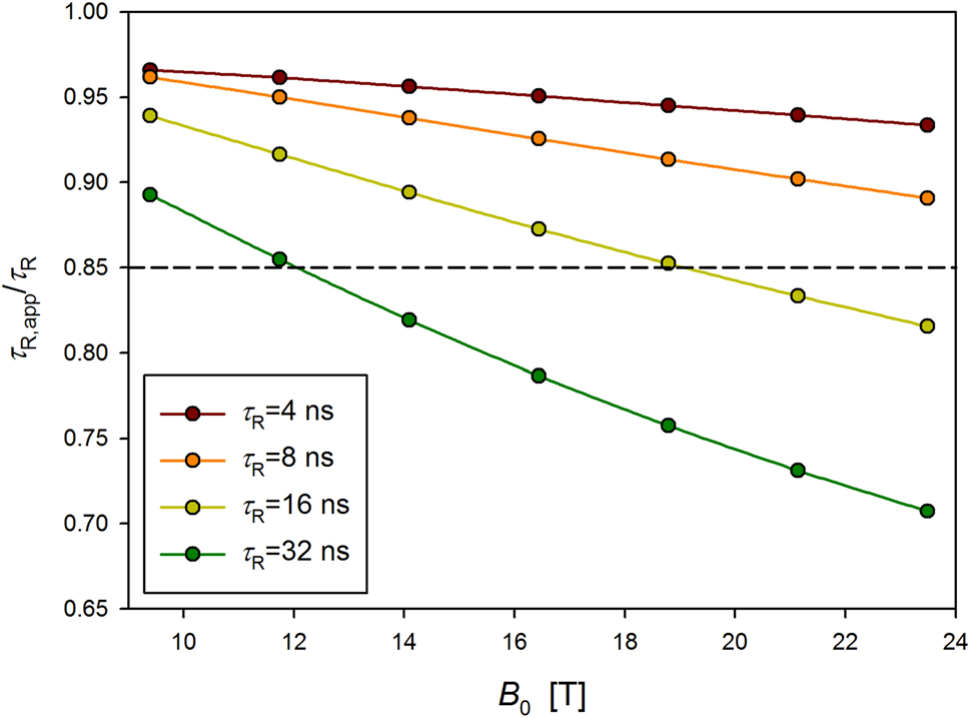
Normalized values of apparent *τ*_R_ evaluated from the *Q* values, given as a fraction of the synthetic *τ*_R_ used in simulations. The *τ*_R,app_/*τ*_R_ ratio is shown as a function of *B*_0_ for several *τ*_R_ values. Calculations were performed applying sizeable internal motion: *S*^2^ = 0.7 and *τ*_int_ = 100 ps. Performance of the *Q*-based method is poor for *B*_0_ and *τ*_R_ corresponding to plots below the dashed line marking 15% deviation of *τ*_R,app_

Use of the *Q* values in the evaluation of an overall correlation time results in the *τ*_R_ underestimation provided the overall tumbling is isotropic (Korzhnev et al. 1997). Influence of the input *τ*_R_ and magnetic field strength values on the value of the apparent *τ*_R_ is demonstrated in Fig. 2. In the utmost situations (parts of plots below the dashed line in the Fig. 2 corresponding to intense internal motion: *S*^2^ = 0.7, *τ*_int_ = 100 ps, slow overall tumbling: *τ*_R_ = 32 ns and very high magnetic field: 23.5 T) the *τ*_R_ evaluation derived from the *Q* values breaks down; relative errors exceed 25%. Use of *Q* values retains the sense only if correlation time of internal motion, *τ*_int_, is short and its amplitude small (Fig. S4).

**Fig. 3:**
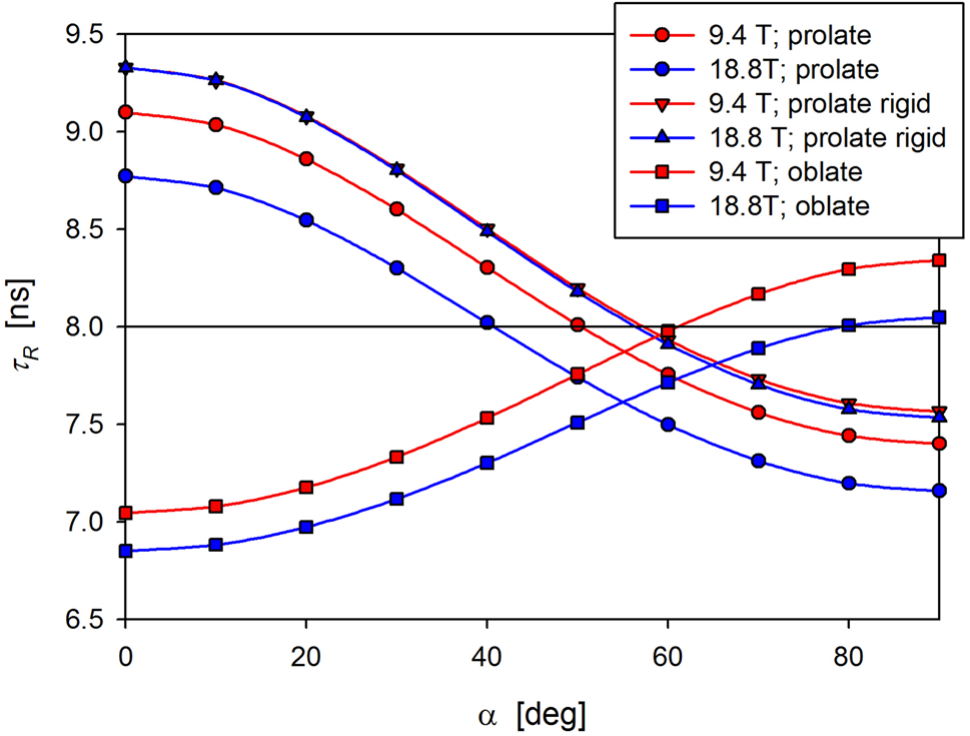
Molecular tumbling is anisotropic (prolate ellipsoid $\left\langle {{\tau _{\text{R}}}} \right\rangle$ = 8 ns, Δ*D* = 1.5 and η = 0). A sizeable internal motion is assumed: *S*^2^ = 0.8 and *τ*_int_ = 100 ps. The *τ*_R_ estimated from the derived *Q* value can deviate significantly from the expected value of 8 ns marked by a horizontal black line. The deviations depend on the N–H vector orientation relative to the unique axis of diffusion tensor given by an angle α. Deviations depend on the magnetic field strength (red and blue circles). Field dependence nearly disappears for rigid N–H vector (*S*^2^ = 1.0; red and blue triangles). The tendency of *τ*_*R*_(α) dependence for oblate ellipsoid ($\left\langle {{\tau _{\text{R}}}} \right\rangle$ = 8 ns, Δ*D* = 0.67 and η = 0) is opposite in comparison with a prolate ellipsoid (red and blue squares)

In the case of anisotropic tumbling, the determined value of the orientation averaged overall correlation time *τ*_R_ = 0.5/(*D*_1_ + *D*_2_ + *D*_3_), can be either larger or smaller than the *τ*_R_ value estimated from *Q* values, depending on the orientation of the N–H vector. Appropriate comparison is presented in Table 1 and Fig. 3.

**Table 1:** Anisotropic tumbling visibly influences on the *Q* and *D* values, while its effect is strongly attenuated regarding *P* values, with variability ranges 25, 19, and 3%, respectively

α [deg]	*R*_1_ [s^−1^]	*R*_2_ [s^−1^]	*Q*	*τ*_R_(*Q*) [ns]	*D* [s^−1^]	*P* [s^−2^]
0	1.983	9.200	4.639	9.098	19.901	26.814
30	2.060	8.791	4.267	8.601	18.819	26.623
60	2.200	8.100	3.682	7.754	16.977	26.203
90	2.263	7.818	3.456	7.401	16.208	25.980

Calculations were performed assuming that anisotropic diffusion tensor is represented by a prolate ellipsoid with the diffusion anisotropy, *ΔD* = 2·*D*_3_/(*D*_1_ + *D*_2_), equal to 1.5, averaged overall correlation time (*τ*_R_) equal to 8 ns and no asymmetry (*η* = |*D*_2_ − *D*_1_|/*D*_3_) since *D*_1_ = *D*_2_. Parameters of internal motion: *S*^2^ = 0.8, *τ*_int_ = 100 ps; *B*_0_ = 9.4 T. The α is an angle between the unique principal axis of the diffusion tensor and N–H vector

Estimation of the average generalized order parameters $S_{{av}}^{2}$ from the experimentally observed *P* values was proposed by Kneller et al. (2002) using formula:

$$S_{{av}}^{2}=\sqrt {\frac{{\left\langle P \right\rangle }}{{{P_{\hbox{max} }}}}} ,$$

where $\left\langle P \right\rangle$ is experimentally observed 10% trimmed mean value and *P*_max_ is determined from the relaxation parameters calculated for a rigid molecule (*S*^2^ = 1.0, *R*_*ex*_ = 0.0) which reorients with *τ*_R_(*Q*). This formula results directly from Eq. (6c). Use of medians is superior to the trimmed mean values since the distributions of *Q, D*, and *P* data are most commonly non gaussian (Table S1) and robust statistics has to be used in their description (Maronna et al. 2006). Medians allow not only avoiding influence of outliers but also eliminating residues from the unstructured segments of protein characterized by inherently small *Q* values and resulting in extremely skewed *Q* distributions. Robust statistics also facilitates identifying residues undergoing chemical exchange as outliers in *Q, D*, and *P* sets. In the following text *Q, D*, and *P* medians are solely used and denoted as $\tilde {Q}$, $\tilde {D}$, and $\tilde {P}$. Therefore, the Kneller et al. formula is rewritten as

7 $$S_{{av}}^{2}=\sqrt {\frac{{\tilde {P}}}{{{P_{\hbox{max} }}}}}$$

This approach is also extended to the estimation of site specific generalized order parameters, $S_{i}^{2}$ utilizing either *D* or *P* values:

8a $$S_{i}^{2}=\frac{{{D_i}}}{{{D_{\hbox{max} }}}}$$

8b $$S_{i}^{2}=\sqrt {\frac{{{P_i}}}{{{P_{\hbox{max} }}}}}$$

**Fig. 4:**
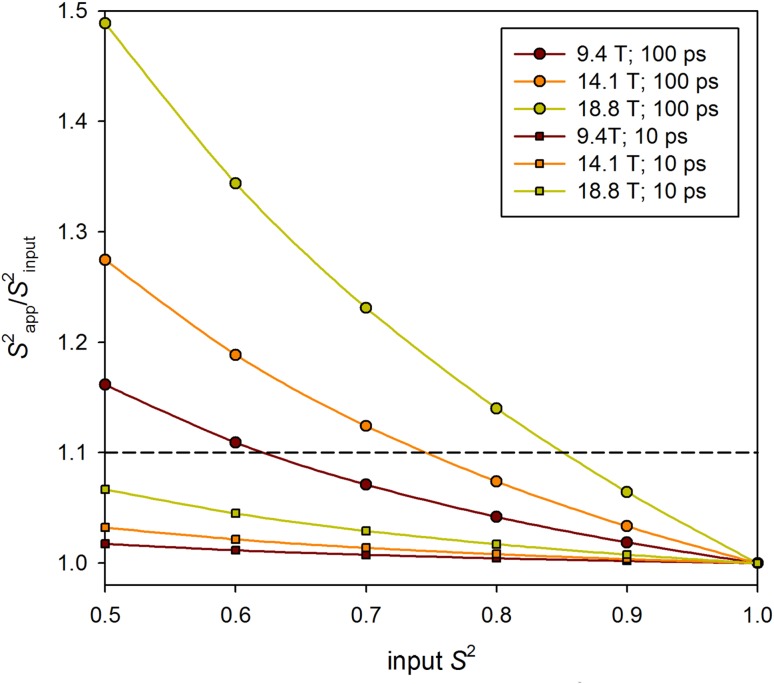
Normalized values of apparent *S*^2^ evaluated from *D* or *P* values (Eqs. 8a and 8b), given as a fraction of the input *S*^2^ used in simulations. The $S_{{app}}^{2}/{S^2}$ ratio is shown as a function of the input *S*^2^ for three *B*_0_ (9.4, 14.1, and 18.8 T) and two *τ*_int_ values (10 and 100 ps). Calculations were performed applying overall correlation time *τ*_*R*_ = 16 ns. Performance of this method becomes poor above the horizontal dashed line representing 10% deviation of the $S_{{app}}^{2}$ values

One has to be aware of possible systematic deviations of so estimated $S_{i}^{2}$ values. As it was shown earlier, the *Q*-derived *τ*_R_ values are underestimated in isotropically tumbling molecules. Accordingly, the *P*_max_ is underestimated as well, resulting in the overestimation of $S_{i}^{2}$ values (Fig. 4). This effect is especially pronounced for slower internal motions (long *τ*_int_) with large amplitudes (small *S*^2^) at high magnetic fields. A similar effect was reported for the relaxation data in ATPase α-domain (Gu et al. 2016).

It is stated that the *Q* values do not distinguish between the effects of motional anisotropy and chemical exchange (Kneller et al. 2002), while the analysis of *P* data significantly attenuates the effects of motional anisotropy (c.f. Table 1) permitting rapid identification of residues undergoing chemical exchange, *R*_*ex*_. It will be shown in the next section, however, that the attenuation of motional anisotropy is not sufficient to identify unequivocally the *R*_*ex*_ influenced residues. The elevated *P* values not always allow identifying residues affected by chemical exchange. In fact, only simultaneous outlying *Q, D*, and *P* values point out unequivocally to the chemical exchange.

## Results and discussion

Combined analysis of *Q, D*, and *P* values is applied to four proteins for which the large relaxation data sets are available in the literature. Essential information on these proteins is collected in the Table 2.

**Table 2:** Basic data concerning GB1, ubiquitin, S100A1, and PSE4 proteins

Protein	GB1	Ubiquitin	S100A1	PSE4
Residues	56	76	2 × 93	271
MW [kDa]	6.3	8.7	21.0	29.3
*τ*_R_ (*dτ*_R_) [ns]	2.05 (0.02)	4.36 (0.03)	8.35 (0.04)	12.30 (0.08)
T [K]	307.0	298.1	310.1	304.6
BMRB code	5569	4245	16360	6838
PDB code	1GB1	1D3Z	2L0P	1G68
Complete MFA results	Table S3	Table S4	Table S5	Table S6
References	Idiyatullin et al. (2003)	Lee and Wand (1999)	Nowakowski et al. (2011)	Morin and Gagné (2009)

### Overall correlation time

The *Q, D*, and *P* site specific values for the analyzed proteins at all available magnetic fields are shown in Figs. 5 and S5–S14.The medians of experimentally observed *Q* = *R*_1_/*R*_2_ values were used for evaluating overall correlation times. Vizualization of this procedure is shown in Figs. S15–S18. Some details are explained in the captions to these figures.

**Fig. 5:**
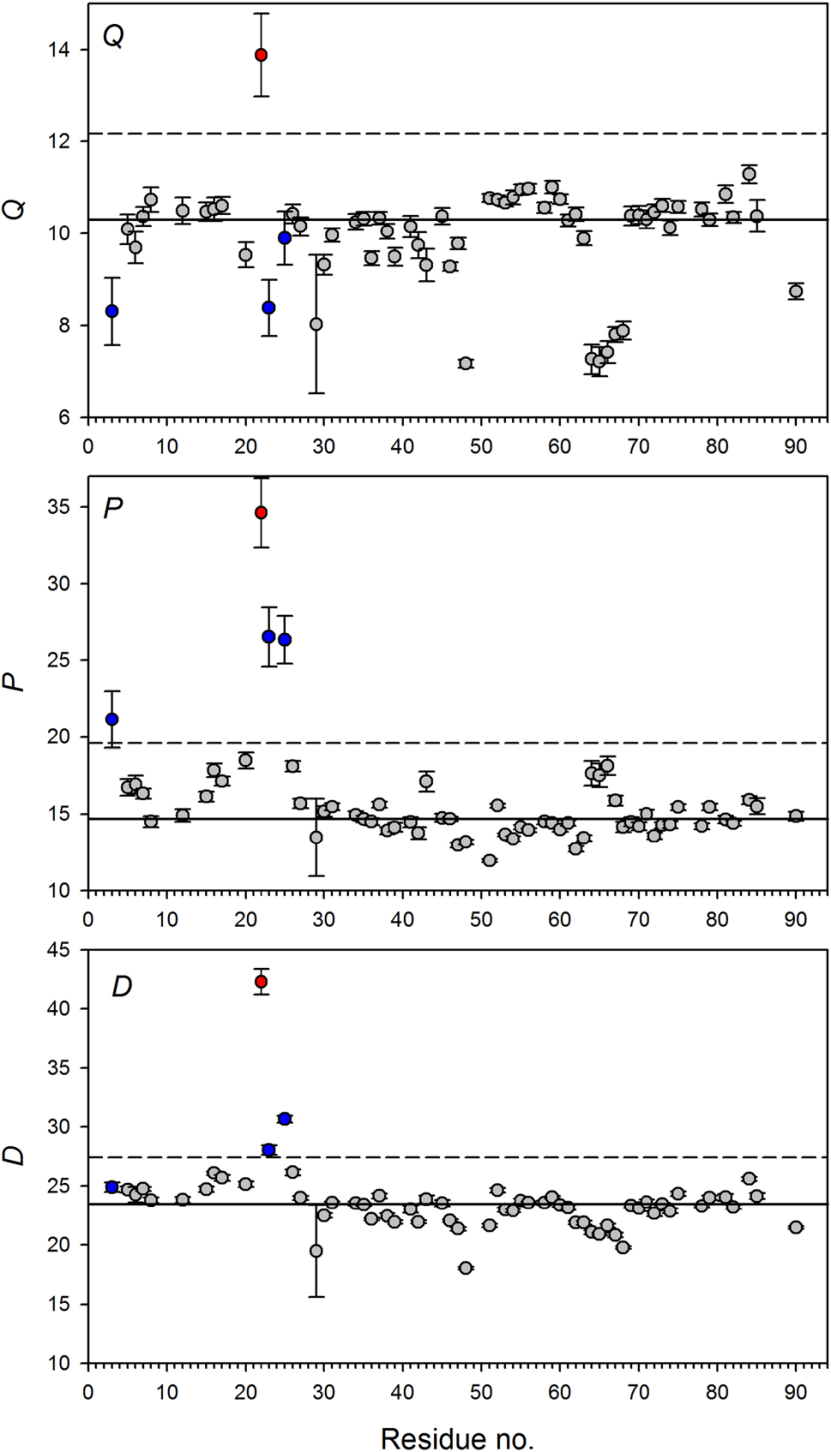
Sequence specific *Q, D*, and *P* values calculated from *R*_1_ and *R*_2_ relaxation rates determined for S100A1 protein at 16.4 T. Solid lines represent medians: $\tilde {Q}$ = 10.31, $\tilde {P}$ = 14.68, and $\tilde {D}$ = 23.49. Dashed lines mark the limit of outliers calculated from the formula *Q*3 + 1.5·*IQR*, where *Q*3 is third quartile and *IQR* is the interquartile range. Residue Glu22 undergoing a chemical exchange is marked with a red circle. Blue circles mark residues with a questionable presence of chemical exchange mechanism

**Fig. 6:**
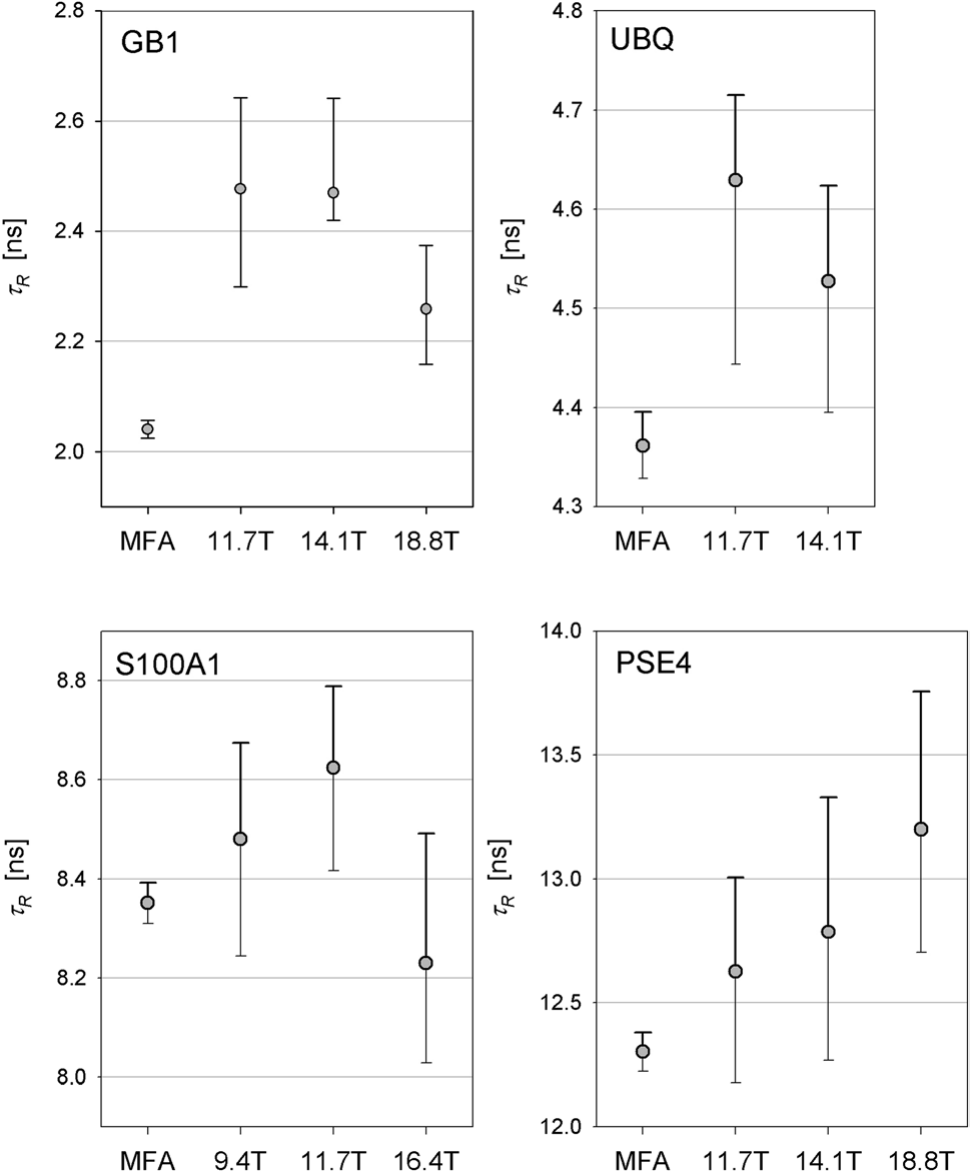
Comparison of the overall correlation times *τ*_R_ determined by the model-free approach with those obtained from the appropriate $\tilde {Q}$ values. Determination of uncertainties represented by error bars is described in the caption to Table S2

For the proteins analyzed here, the determined *τ*_R_(*Q*) values are larger than the corresponding values obtained from the MFA analysis as shown in the Fig. 6. Corresponding numerical values are reported in Table S2. The averaged deviations are equal to 18, 5, 2, and 5% for GB1, ubiquitin, S100A1, and PSE4, respectively. The opposite results would be expected from the simulation shown in Fig. 2. However, it is demonstrated in the “Method” section that the anisotropic tumbling can result in the overestimation of *τ*_R_ values and one should note that all four proteins reorient anisotropically (Table 3). It is worth noting that the largest discrepancy between *Q*-based and MFA derived *τ*_R_ values appears for GB1 characterized by the strongest tumbling anisotropy. Anisotropic tumbling and, therefore, the possible overestimation of *τ*_R_ values can be anticipated from the ratios of principal values of inertia tensor provided the protein structure is available (Mandel et al. 1995).

**Table 3:** Anisotropic tumbling of the analyzed proteins

Protein	GB1	Ubq	S100A1	PSE4
*τ*_R_ [ns]^a^	2.05	4.36	8.35	12.30
*D*_1_ [10^7^ s^−1^]	7.29	3.23	2.12	1.31
*D*_2_ [10^7^ s^−1^]	7.35	3.76	2.10	1.17
*D*_3_ [10^7^ s^−1^]	9.87	4.47	1.77	1.59
Δ*D*^b^	1.35 (0.02)	1.28 (0.02)	0.84 (0.01)	1.28 (0.01)
*η*	0.006	0.119	0.035	0.084
I_1_:I_2_:I_3_^c^	0.91:1.00:0.52^d^	0.90:1.00:0.62^d^	0.65:0.74:1.00^e^	1.00:0.89:0.58^d^
Δ*D*_pred_^f^	1.36	1.24	0.83	1.28

Isotropic correlation time: *τ*_R_ = 0.5/(*D*_1_ + *D*_2_ + *D*_3_), anisotropy: *ΔD* = 2·*D*_3_/(*D*_1_ + *D*_2_), asymmetry: *η* = |*D*_2_ − *D*_1_|/*D*_3_

^a^The MFA analysis was performed for all available relaxation data disregarding the results reported in original papers; the details of the calculations and their results including uncertainties of parameters, are given in the Tables S3–S6

^b^Uncertainties of anisotropies were calculated from the diffusion constants uncertainties applying a standard method of error propagation

^c^The principal value ratios of the inertia tensors calculated from the PDB structures (c.f., Table 1)

^d^Prolate ellipsoid: I_3_ < I_1_, I_2_, Δ*D* > 1.0

^e^Oblate ellipsoid: I_3_ > I_1_, I_2_, Δ*D* < 1.0

^f^Evaluation of the diffusion anisotropy performed with the formula Δ*D*_pred_ = [(I_1_ + I_2_)/2I_3_]^1/2^

Anisotropic tumbling as a reason of the divergence between *τ*_R_ values obtained from the MFA analysis and *Q*-based method can be demonstrated analyzing two residues of GB1, Thr17 and Asp40. Their N–H vectors are nearly perpendicular one another. The *Q* values have been obtained from the *R*_1_ and *R*_2_ relaxation rates and next used in the *τ*_*R*_(*Q*) determination. Results are presented in Table 4. The orientation of N–H vectors strongly influences the value of pseudo-isotropic overall correlation time determined using *Q* value. It has to be pointed out that the median, $\tilde {Q}$, can be skewed due to the non uniform distribution of N–H vector orientations. Regardless of anisotropic tumbling the presented examples of *τ*_R_ estimation by means of the *Q*-based method give suitable results with the average deviation 7% and the largest one 21% for GB1 at 11.7 T as compared with the MFA results.

**Table 4:** Overall correlation times *τ*_R_ determined for GB1 protein from the experimentally derived *Q* values

*B*_0_ [T]	$\tilde {Q}$	*τ*_*R*_($\tilde {Q}$)	*Q* Thr17	*τ*_*R*_(*Q*) Thr17	*Q* Asp40	*τ*_*R*_(*Q*) Asp40
11.7	1.54 (0.06)	2.48 (0.17)	1.41 (0.11)	2.04 (0.41)	1.80 (0.14)	3.16 (0.32)
14.1	1.72 (0.05)	2.47 (0.11)	1.70 (0.11)	2.42 (0.24)	1.85 (0.09)	2.72 (0.18)
18.8	2.01 (0.08)	2.26 (0.11)	2.01 (0.07)	2.12 (0.10)	2.10 (0.08)	2.36 (0.10)

$\tilde {Q}$—the median of the *Q* value set for all available residues of GB1, *Q*(Thr17), *Q*(Asp40)—individual residues. Angle between N–H vectors—83°

### Chemical exchange

Residues exhibiting slow conformational mobility in the micro- to millisecond time scale have to be selected and next skipped in the further analysis of fast motions. Elevated *Q, P*, or *D* values owing to the chemical exchange mechanism result in non physical values of the generalized order parameter, *S*^2^ > 1.

None of GB1 residues shows outlying *Q, P*, or *D* values being a hallmark of the chemical exchange (c.f. Figs. S5–S7). This observation is consistent with the MFA analysis performed for *R*_1_, *R*_2_, and *NOE* data at three magnetic fields (Table S3). Both, standard deviations of Φ factors multiplied by an appropriate Student’s *t*-value and F-test applied to the partial target functions characterizing the fit quality for a given residue with and without assumption of chemical exchange (three or two local parameters) were applied to verify the significance of the MFA-derived Φ factors.

Among the *Q, P*, and *D* values calculated for ubiquitin one residue, Asn25, displays the largest values pointing out to the effective chemical exchange mechanism in the relaxation of this residue (c.f. Figs. S8 and S9). This result is consistent with the results of the MFA analysis. Asn25 is the unique residue exhibiting meaningful Φ value (Table S4). Three other residues show large but erratic *Q, P, D* values. None of them, however, possesses a meaningful Φ value in the MFA analysis.

Glu22 is the unique residue in the S100A1 protein displaying simultaneous large *Q, P*, and *D* values, thus clearly exhibiting the chemical exchange mechanism (Figs. 5, S10, and S11). This fact is also consistent with the MFA analysis (Table S5). However, three other residues, Glu3, Gly23 (only at 16.4 T), and Lys25, similarly as Glu22, display distinct *P* values. So, they can be suspected to undergo a chemical exchange. On the other hand, their *Q* values and majority of *D* values are very close to the corresponding medians. Φ factors of Gly23 and Lys25 residues obtained in the MFA analysis reveal the chemical exchange mechanism for these residues but definitely exclude it for Glu3 residue. Summing up, *Q* values fail to recognize chemical exchange for Gly23 and Lys25 but *P* values can lead to false recognition of non existing slow motions. *D* values perform the best at 16.4 T but fail at 9.4 T. None of *Q, P*, and *D* parameters is fully immune to the erroneous identification of chemical exchange.

In PSE4 protein (Figs. S12–S14) *Q, P, D* values for two residues, Thr128 and Ser235, at 11.7 T exceed corresponding outliers’ limits indicating chemical exchange. They also display the largest Φ values in the MFA analysis (Table S6). Nevertheless, not all of *Q, P, D* values at two higher magnetic fields confirm the efficient chemical exchange. Several other residues show non systematic, outlying *Q, P, D* values. Three of them, Thr57, Leu221, and Gly236, exhibit meaningful Φ values indicating chemical exchange while for the remaining residues, protruding *Q, P*, or *D* values seem to be misleading.

All the above discussed results point out to the possibility of ambiguous or false recognition of chemical exchange involved residues. None of *Q, P*, and *D* parameters is fully immune to the erroneous identification of chemical exchange but their values inspected simultaneously usually identify residues influenced by chemical exchange with a high probability.

### Generalized order parameters

There are three methods allowing to estimate site specific generalized order parameters *S*^2^ which describe local fast mobility of a protein backbone. Since such information is important in study of protein function and interactions with other molecules, it is necessary to compare results of these methods. Determination of the isotropic, overall tumbling correlation time, *τ*_*R*_, from the properly selected *Q* value makes a starting point of all these methods. Two methods utilizing Eqs. (8a) or (8b) are based on *D* or *P* values, respectively. The third method, LMFA, requires fit of back calculated relaxation rates *R*_1_ and *R*_2_ with fixed *τ*_*R*_(*Q*) and adjustable *S*^2^ parameters to the experimental data.

**Fig. 7:**
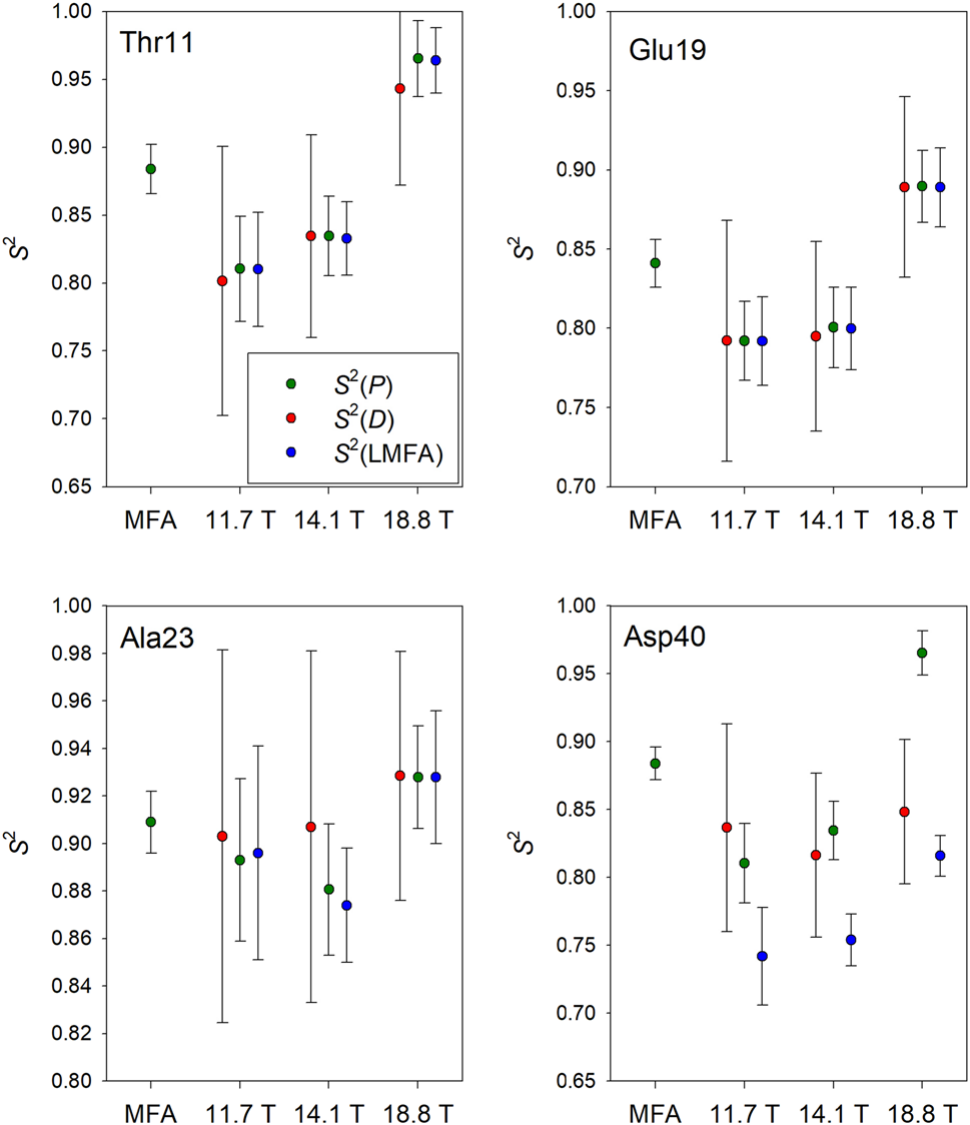
Comparison of *S*^2^ values and their confidence limits for selected residues of GB1 protein. *S*^2^ obtained from *D* values, *P* values, and LMFA approach are represented by red, green, and blue circles, respectively. The leftmost values are reference MFA derived results. Confidence limits for the MFA and LMFA calculations were obtained as standard deviations from 200 Monte Carlo simulations. Symmetrized confidence limits for *S*^2^(*D*) and *S*^2^(*P*) values were evaluated applying standard method of error propagation

Comparison of the results of these methods with those obtained for the MFA analysis of all available relaxation data as a reference, was performed. Similar conclusions can be reached from the results obtained for each of four investigated proteins. General tendencies can be discussed and presented on the examples of several selected GB1 residues. They are shown in Fig. 7.

An important conclusion drawn from data shown in Fig. 7 is that confidence ranges of *S*^2^(*D*) are roughly twice larger than corresponding ranges for *S*^2^(*P*) and *S*^2^(LMFA). This is a general feature obtained for all residues in all analyzed proteins (total 399 residues). Confidence ranges for *S*^2^(*P*) and *S*^2^(LMFA) are comparable, but always larger than those characterizing MFA results for obvious reason of a larger number of data and parameters used in the latter method. Another important factor concerns dispersion of *S*^2^ values resulting from different methods for the same residue. In the majority of cases *S*^2^(*D*), *S*^2^(*P*), and *S*^2^(LMFA) are close each other at a given magnetic field but differ from the *S*^2^(MFA) and among the data determined at different magnetic fields (Fig. 7, Thr11 and Glu19). In a number of cases all the data are close each other (Fig. 7, Ala23). Such situation takes place when all individual *Q* values are close to the corresponding medians $\tilde {Q}$. In the opposite case, when individual *Q* values differ markedly from medians, all *S*^2^ values are broadly dispersed (Fig. 7, Asp40).

Whole sets of *S*^2^ values estimated as the sums of squared differences between *S*^2^(MFA) and *S*^2^(*X*) (*X* = *D, P*, LMFA) allow to estimate accuracy of each method. Once more *P*-based and LMFA approaches are comparable and slightly more accurate than the *D*-based one.

Concluding, *P*-based and LMFA methods of *S*^2^ determination perform similarly but the former one is less demanding from the computational standpoint.

## Conclusions

The *R*_1_ and *R*_2_ relaxation rates of ^15^N nuclei measured at a single magnetic field strength in proteins are not sufficient to perform a formal MFA analysis, but can be utilized for the semi-quantitative evaluation of the overall tumbling correlation time from the median of the set of transverse to longitudinal relaxation rates *Q* = *R*_2_/*R*_1_. Generalized order parameters *S*^2^ characterizing amplitude of internal local motions, faster than the overall tumbling, can be site selectively evaluated using either linear combination *D* = 2*R*_2_ − *R*_1_ or product *P* = *R*_2_·*R*_1_ or LMFA method. Efficiency of these methods was carefully compared and the *P* and LMFA methods are comparably accurate while the former is less demanding from the computational standpoint. As the final result one obtains estimation of the overall correlation time, *τ*_R_, and parameters characterizing internal motions, *S*^2^, on the time scales faster than *τ*_R_. Additionally, residues undergoing conformational motions in the micro- to millisecond time scale can be selected basing on the *Q, D*, and *P* outliers.

## Electronic supplementary material

Below is the link to the electronic supplementary material.

Supplementary material 1 (PDF 1915 KB)
