# Supplementary material for: Fast evaluation of protein dynamics from deficient 15N relaxation data
Source: J Biomol NMR. 2018 Mar 28;70(4):219–28. doi: 10.1007/s10858-018-0176-3 (PMC5953972; doi:10.1007/s10858-018-0176-3)
Supplement: Supplementary file 1 — Supplementary material 1 (PDF 1915 KB) [file 10858_2018_176_MOESM1_ESM.pdf]

Fast evaluation of protein dynamics from deficient ^{15}N relaxation data

Łukasz Jaremko[1], Mariusz Jaremko[1], Andrzej Ejchart[2], Michał Nowakowski[3*]

[1] *Division of Biological and Environmental Sciences and Engineering, King Abdullah University of Science and Technology (KAUST), Thuwal 23955-6900, Kingdom of Saudi Arabia*
[2] *Institute of Biochemistry and Biophysics, Polish Academy of Science, Laboratory of Biological NMR, Pawinskiego 5A, 02-106 Warszawa, Poland*
[3] *Faculty of Chemistry, Biological and Chemical Research Centre, University of Warsaw, Żwirki i Wigury 101, 02-089 Warszawa, Poland*
[*] to whom correspondence should be addressed
lyam@chem.uw.edu.pl

Table of Contents:

Pages 2-19 SI-Figures
Pages 20-31 SI-Tables

Figure S1

Contour plot presenting Q values normalized in relation to the Q in rigid molecule. τ_R=10 ns and B_0=14.1 T were used in calculations.

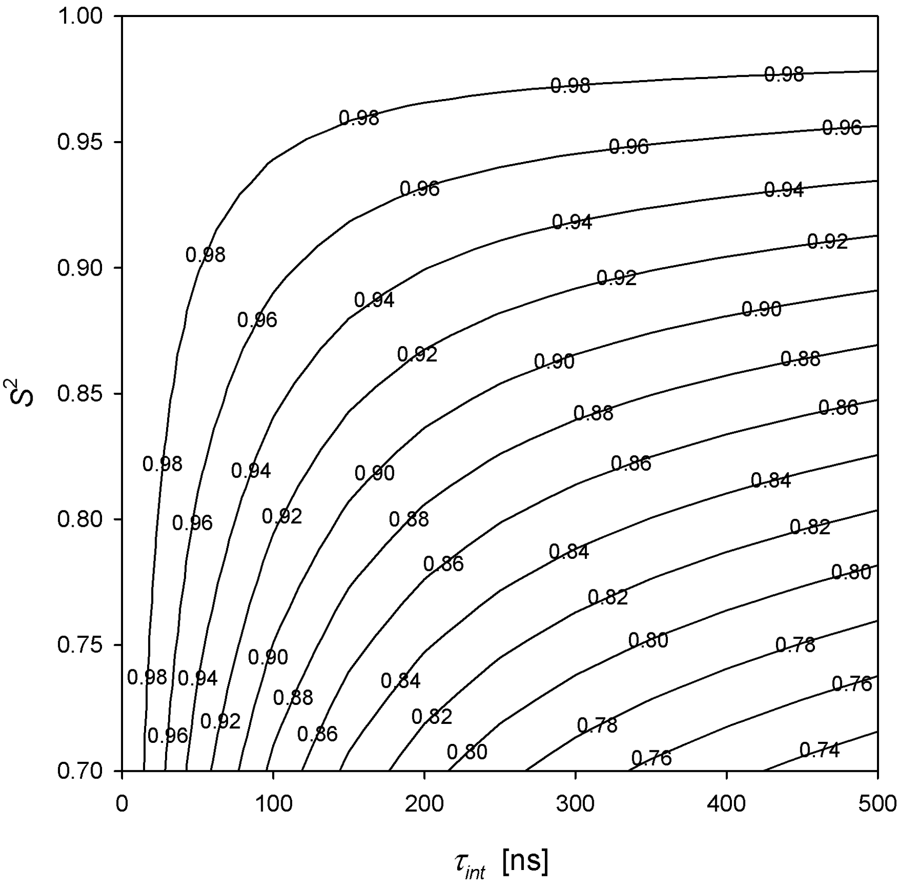

Figure S2

Contour plot presenting *P* values normalized in relation to the *P* in rigid molecule. τ_R=10 ns and B_0=14.1 T were used in calculations.

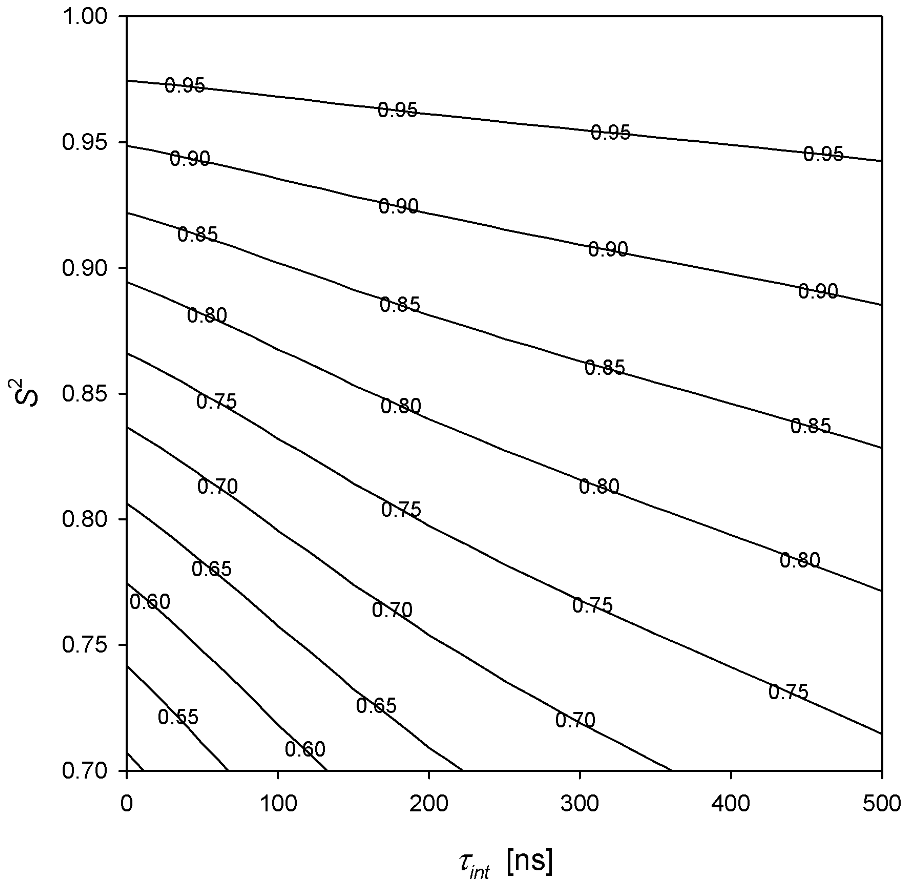

Figure S3

Contour plot presenting *D* values normalized in relation to the *D* in rigid molecule. τ_R=10 ns and B_0=14.1 T were used in calculations.

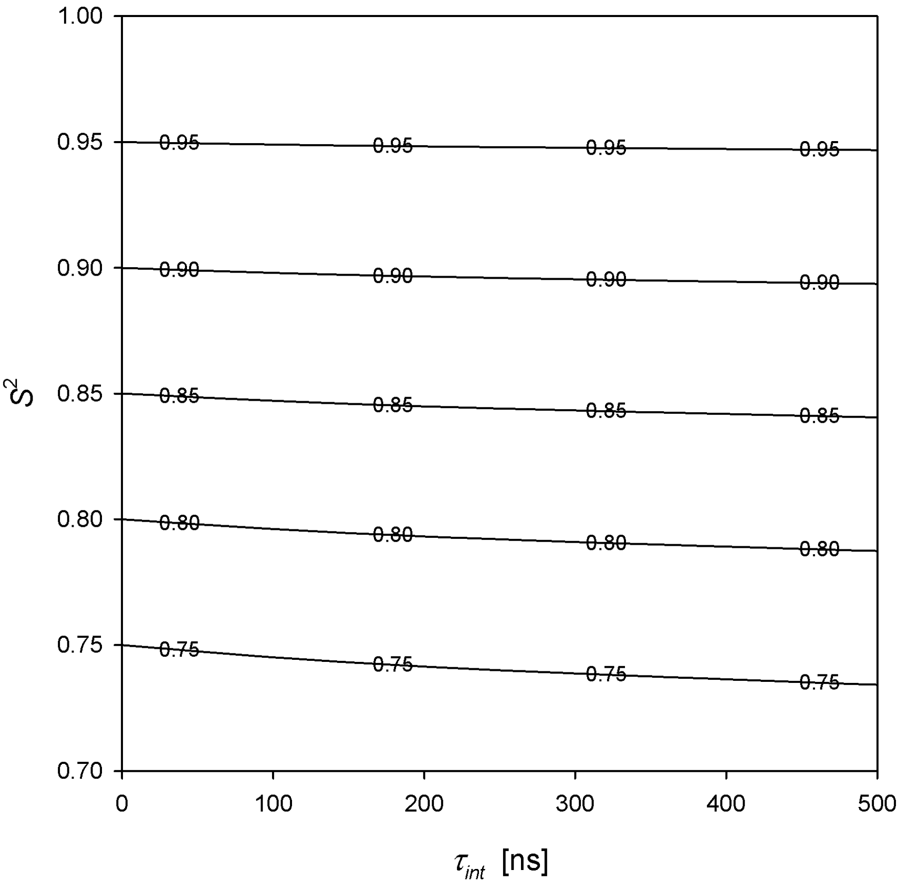

Figure S4

Contour plot presenting underestimation of the apparent τ_R values determined from the $Q = R_2/R_1$ ratio as a function of internal motion parameters, normalized in relation to the synthetic τ_R=10 ns at B_0=18.8 T.

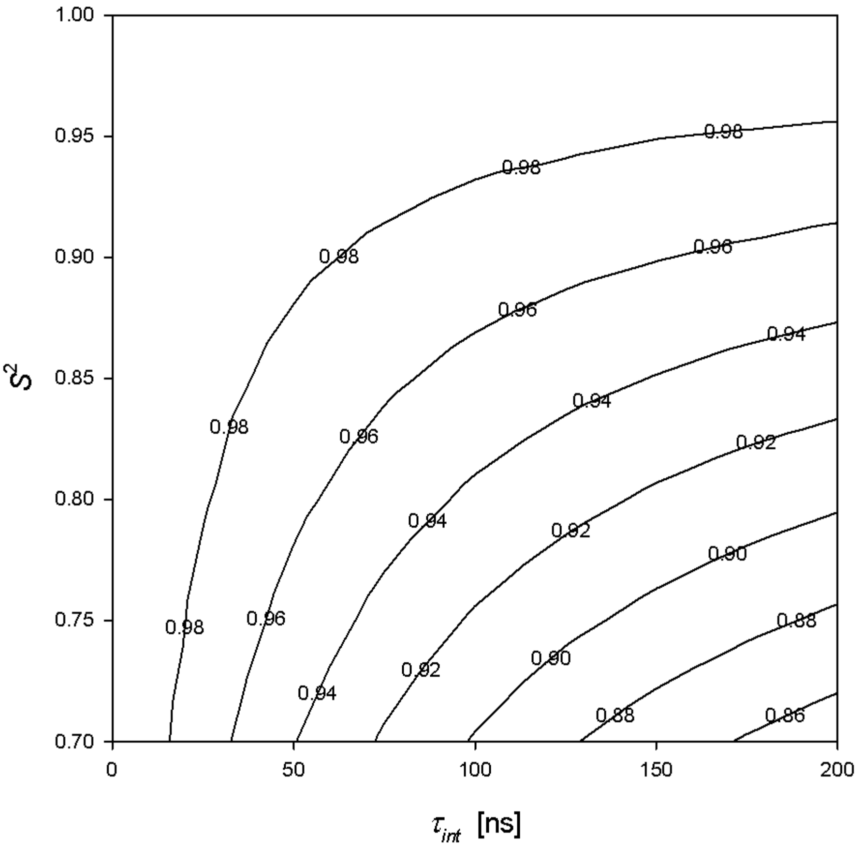

Figure S5
Sequence specific *Q*, *P*, and *D* values calculated from R_1 and R_2 relaxation rates determined for GB1 protein at 11.7 T. Solid lines represent medians: $\tilde{Q}$ =1.54, $\tilde{P}$ =8.92, and $\tilde{D}$ =4.99. Dashed lines mark the limit of outliers calculated from the formula $Q3 + 1.5 \cdot IQR$, where $Q3$ is third quartile and IQR is interquartile range.

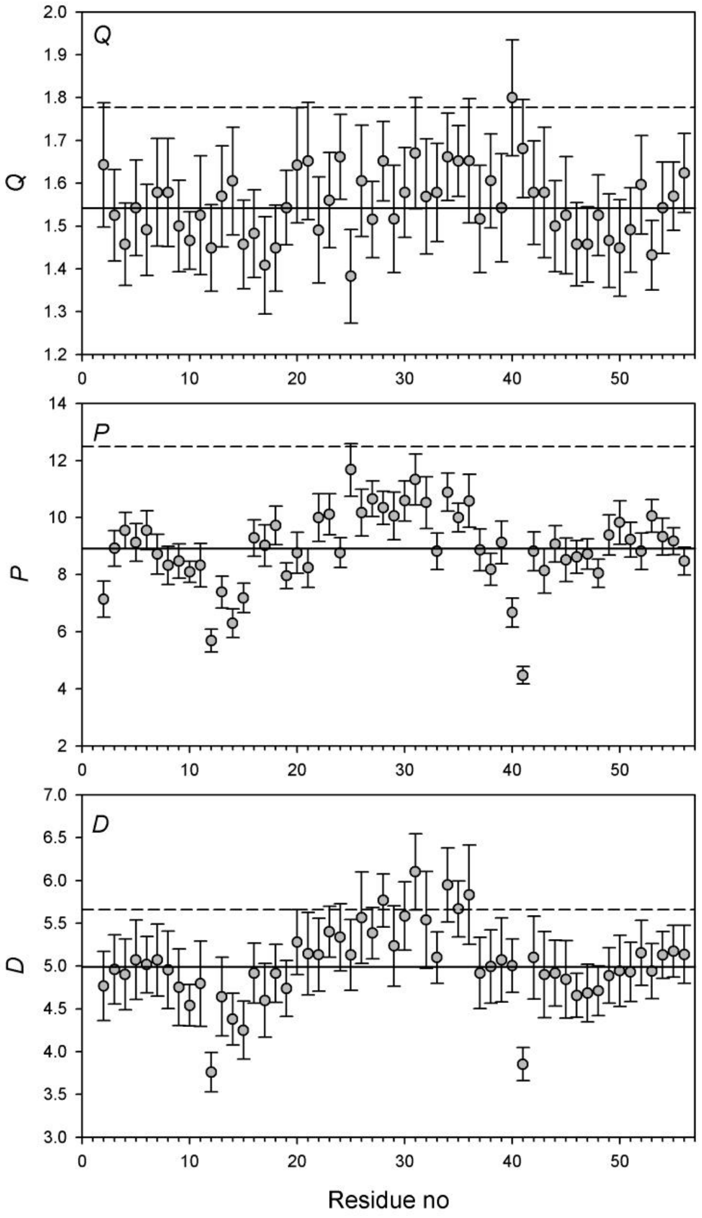

Figure S6

Sequence specific *Q*, *P*, and *D* values calculated from R_1 and R_2 relaxation rates determined for GB1 protein at 14.1 T. Solid lines represent medians: $\tilde{Q}$ =1.72, $\tilde{P}$ =9.18, and $\tilde{D}$ =5.65. Dashed lines mark the limit of outliers calculated from the formula $Q3 + 1.5 \cdot IQR$, where $Q3$ is third quartile and *IQR* is interquartile range.

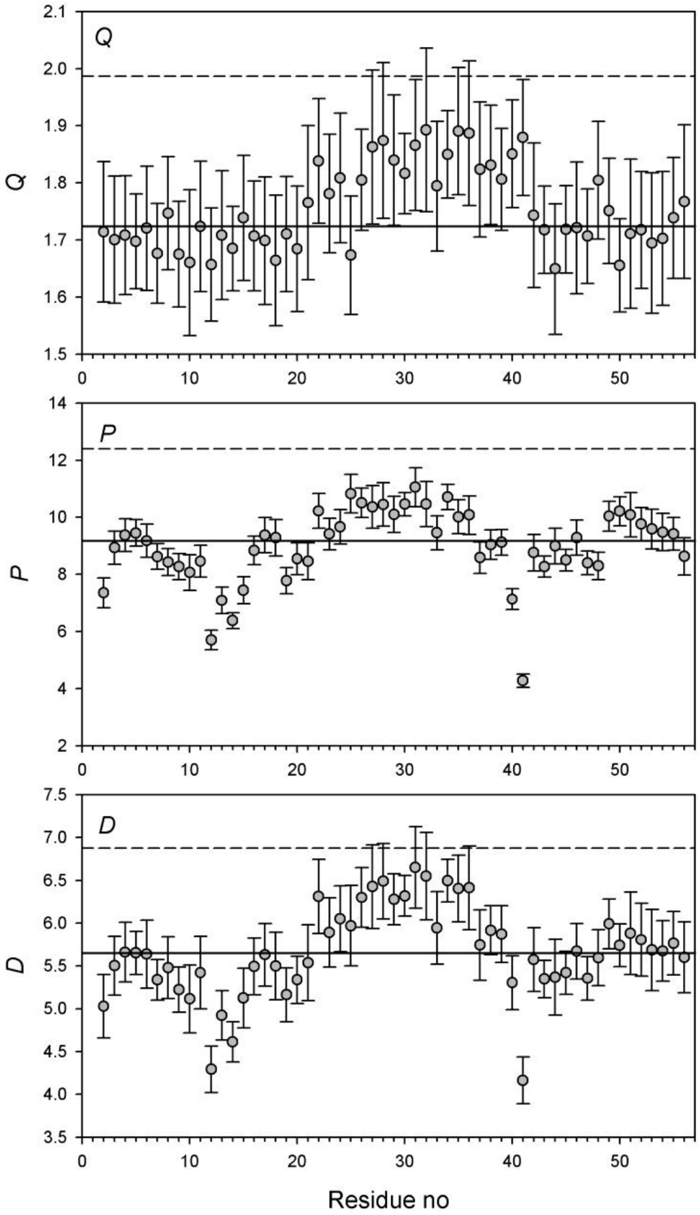

Figure S7
Sequence specific *Q*, *P*, and *D* values calculated from R_1 and R_2 relaxation rates determined for GB1 protein at 18.8 T. Solid lines represent medians: $\tilde{Q}$ =2.02, $\tilde{P}$ =9.63, and $\tilde{D}$ =6.66. Dashed lines mark the limit of outliers calculated from the formula $Q3 + 1.5 \cdot IQR$, where $Q3$ is third quartile and IQR is interquartile range.

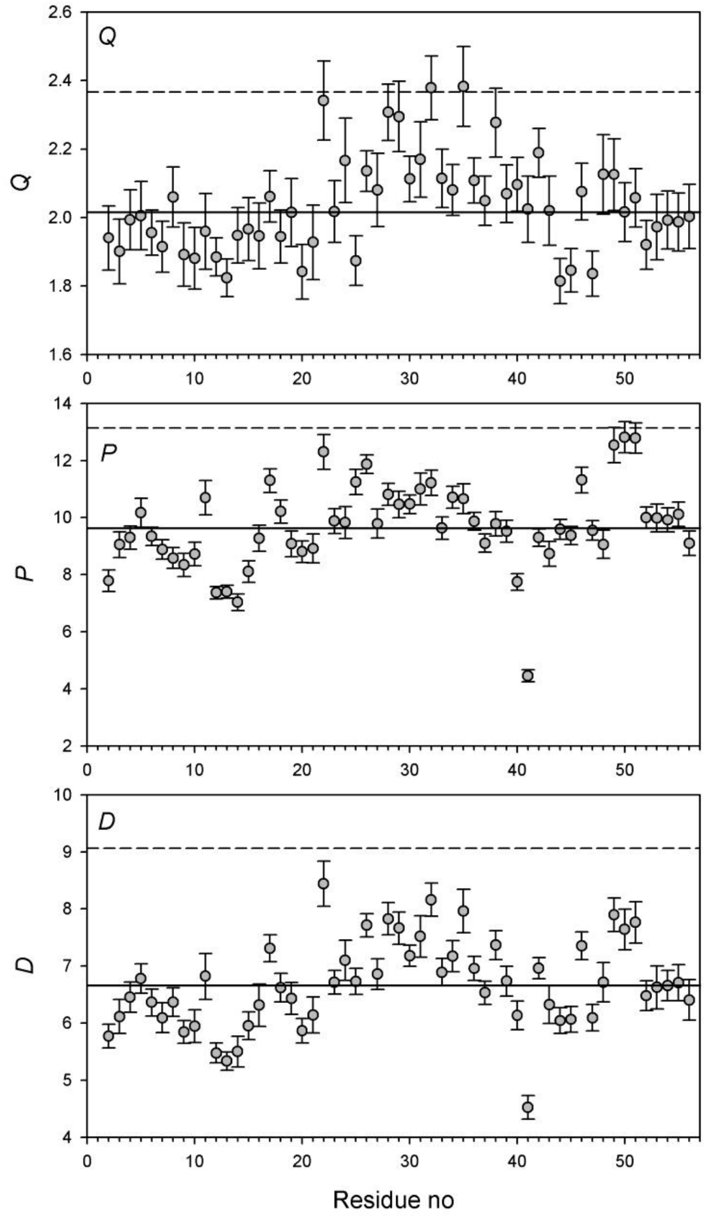

Figure S8

Sequence specific *Q*, *P*, and *D* values calculated from R_1 and R_2 relaxation rates determined for ubiquitin at 11.7 T. Solid lines represent medians: $\tilde{Q}$ =2.55, $\tilde{P}$ =14.75, and $\tilde{D}$ =9.81. Dashed lines mark the limit of outliers calculated from the formula *Q*3 + 1.5·*IQR*, where *Q*3 is third quartile and *IQR* is interquartile range. Residue Asn25 undergoing a chemical exchange is marked with a red circle. Blue circles mark residues with a questionable presence of chemical exchange mechanism.

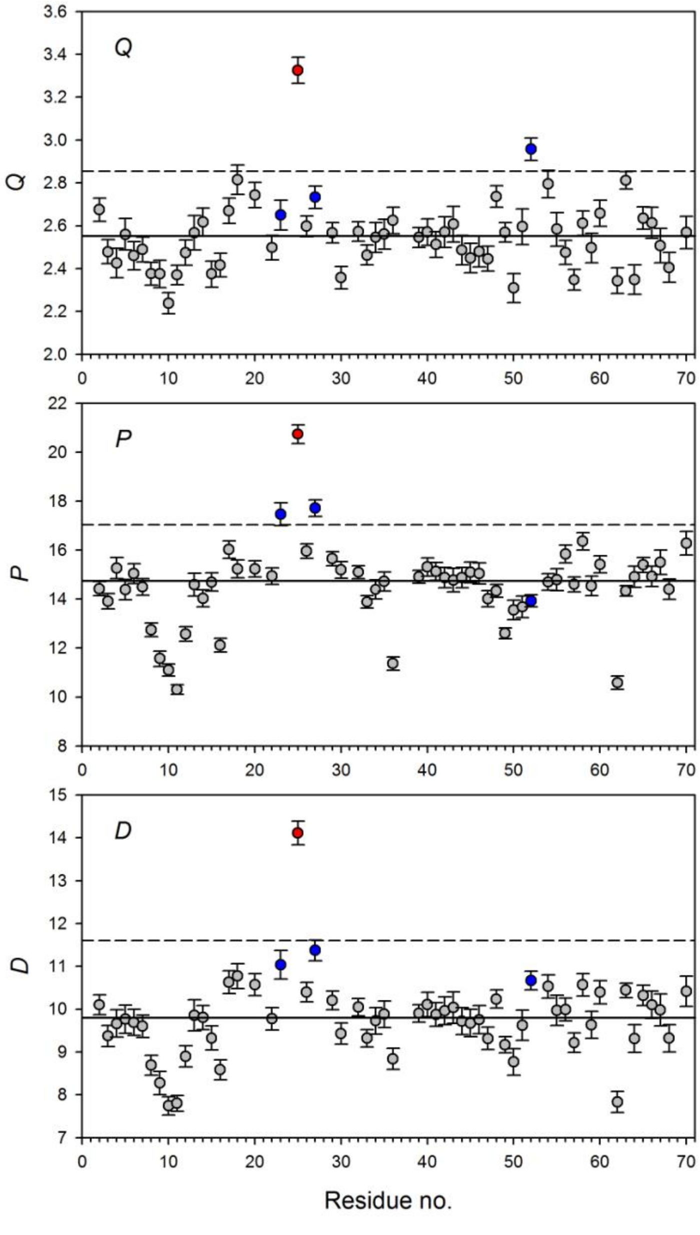

Figure S9

Sequence specific Q, P, and D values calculated from R_1 and R_2 relaxation rates determined for ubiquitin at 14.1 T. Solid lines represent medians: $\tilde{Q}$ =3.10, $\tilde{P}$ =12.90, and $\tilde{D}$ =10.69. Dashed lines mark the limit of outliers calculated from the formula $Q3 + 1.5 \cdot IQR$, where $Q3$ is third quartile and IQR is interquartile range. Residue Asn25 undergoing a chemical exchange is marked with a red circle. Blue circles mark residues with a questionable presence of chemical exchange mechanism.

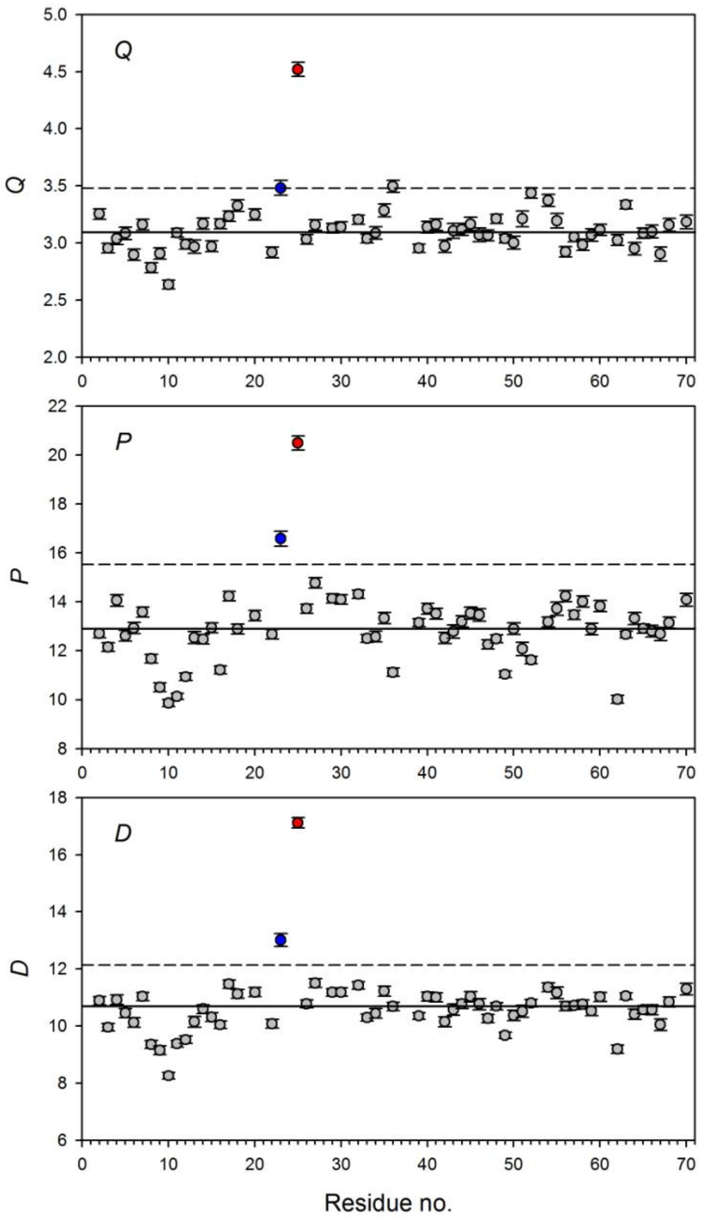

Figure S10
Sequence specific Q, P, and D values calculated from R_1 and R_2 relaxation rates determined for S100A1 protein at 9.4 T. Solid lines represent medians: $\tilde{Q}$ =4.18, $\tilde{P}$ =24.13, and $\tilde{D}$ =17.56. Dashed lines mark the limit of outliers calculated from the formula $Q3 + 1.5 \cdot IQR$, where $Q3$ is third quartile and IQR is interquartile range. Residue Glu22 undergoing a chemical exchange is marked with a red circle. Blue circles mark residues with a questionable presence of chemical exchange mechanism.

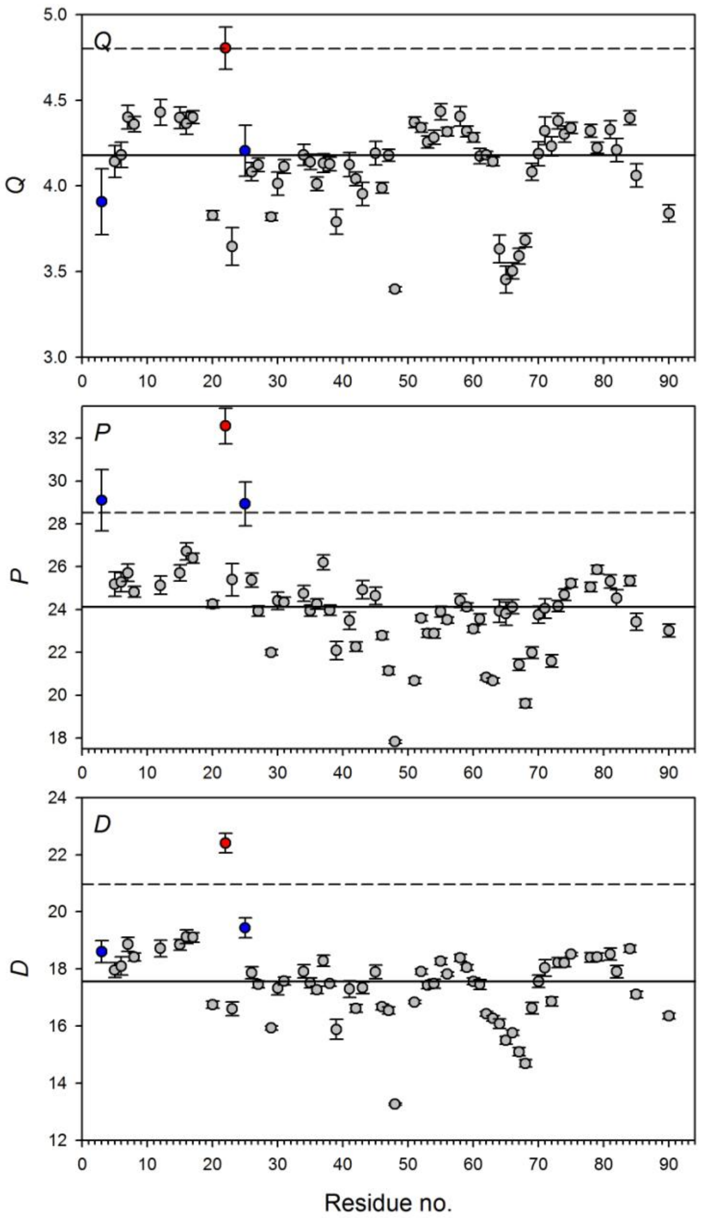

Figure S11
Sequence specific *Q*, *P*, and *D* values calculated from R_1 and R_2 relaxation rates determined for S100A1 protein at 11.7 T. Solid lines represent medians: $\tilde{Q}$ =6.06, $\tilde{P}$ =18.01, and $\tilde{D}$ =19.23. Dashed lines mark the limit of outliers calculated from the formula $Q3 + 1.5 \cdot IQR$, where *Q*3 is third quartile and *IQR* is interquartile range. Residue Glu22 undergoing a chemical exchange is marked with a red circle. Blue circles mark residues with a questionable presence of chemical exchange mechanism.

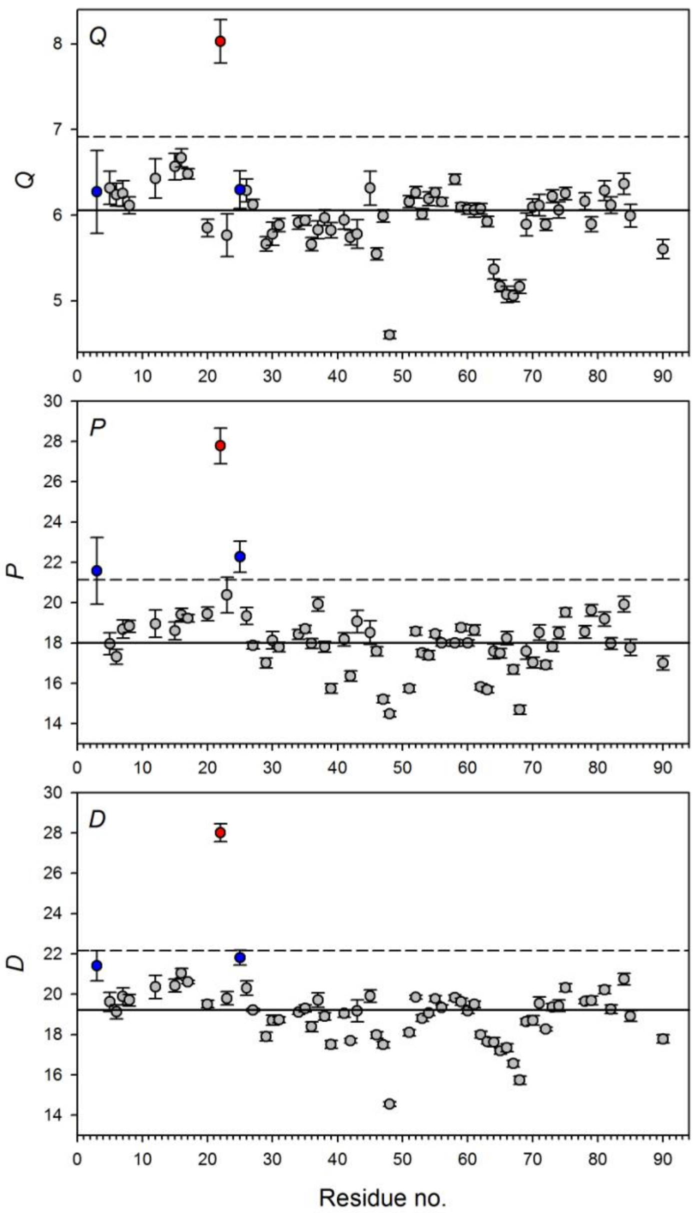

Figure S12
Sequence specific *Q*, *P*, and *D* values calculated from R_1 and R_2 relaxation rates determined for PSE4 protein at 11.7 T. Solid lines represent medians: $\tilde{Q}$ =11.68, $\tilde{P}$ =20.56, and $\tilde{D}$ =29.80. Dashed lines mark the limit of outliers calculated from the formula $Q3 + 1.5 \cdot IQR$, where $Q3$ is third quartile and IQR is interquartile range. Residues undergoing a chemical exchange are marked with a red circles. Blue circles mark residues with a questionable presence of chemical exchange mechanism.

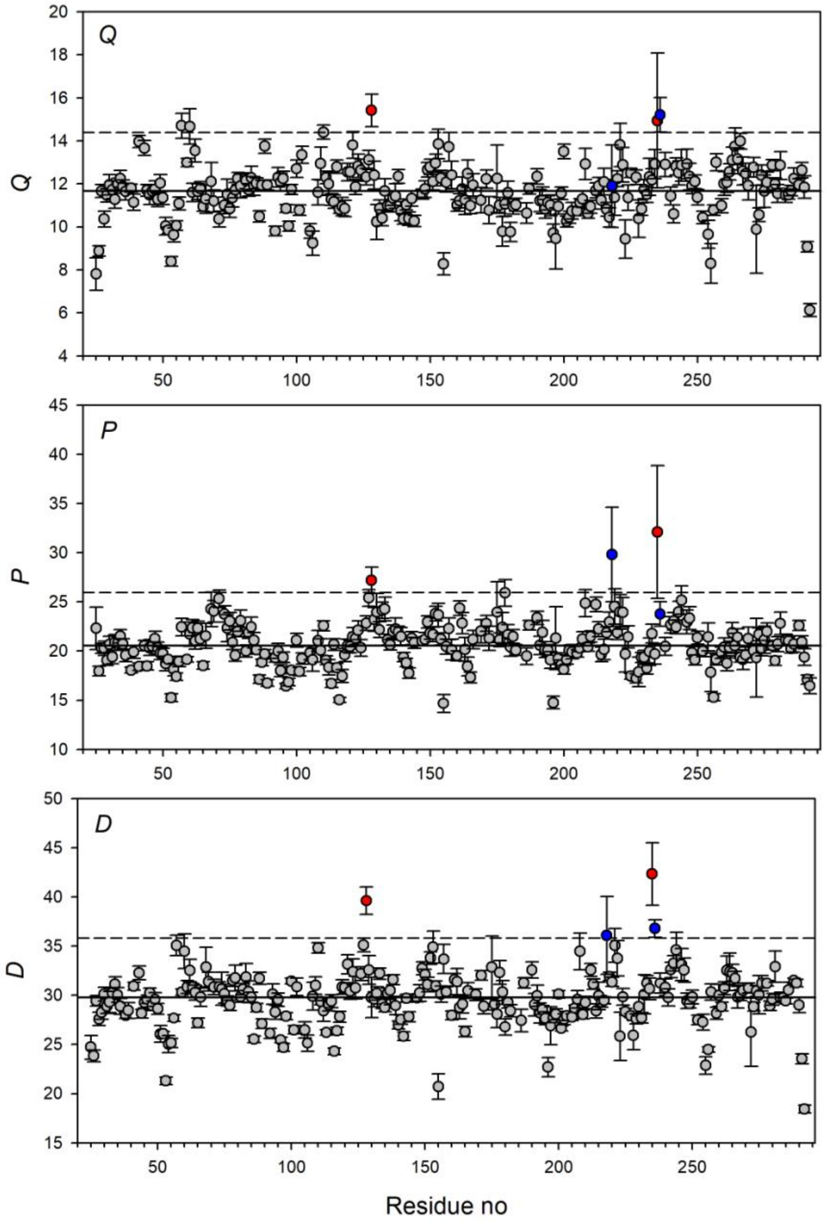

Figure S13

Sequence specific Q, P, and D values calculated from R_1 and R_2 relaxation rates determined for PSE4 protein at 14.1 T. Solid lines represent medians: $\tilde{Q}$ =16.74, $\tilde{P}$ =16.27, and $\tilde{D}$ =32.20. Dashed lines mark the limit of outliers calculated from the formula $Q3 + 1.5 \cdot IQR$, where $Q3$ is third quartile and IQR is interquartile range. No residues undergoing an unequivocal chemical exchange can be identified. Blue circles mark residues with a questionable presence of chemical exchange mechanism.

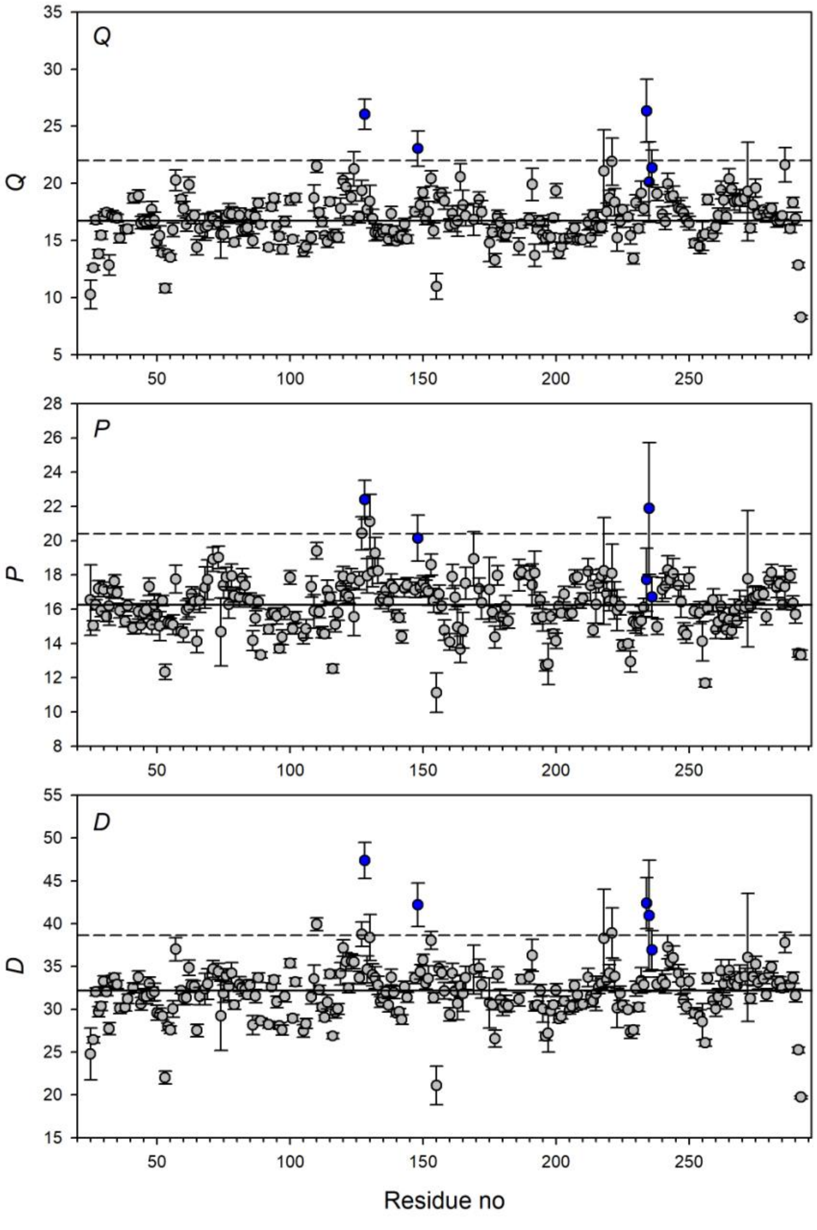

Figure S14

Sequence specific Q, P, and D values calculated from R_1 and R_2 relaxation rates determined for PSE4 protein at 18.8 T. Solid lines represent medians: $\tilde{Q}$ =30.80, $\tilde{P}$ =13.79, and $\tilde{D}$ =40.48. Dashed lines mark the limit of outliers calculated from the formula $Q3 + 1.5 \cdot IQR$, where $Q3$ is third quartile and IQR is interquartile range. No residues undergoing an unequivocal chemical exchange can be identified. Blue circles mark residues with a questionable presence of chemical exchange mechanism.

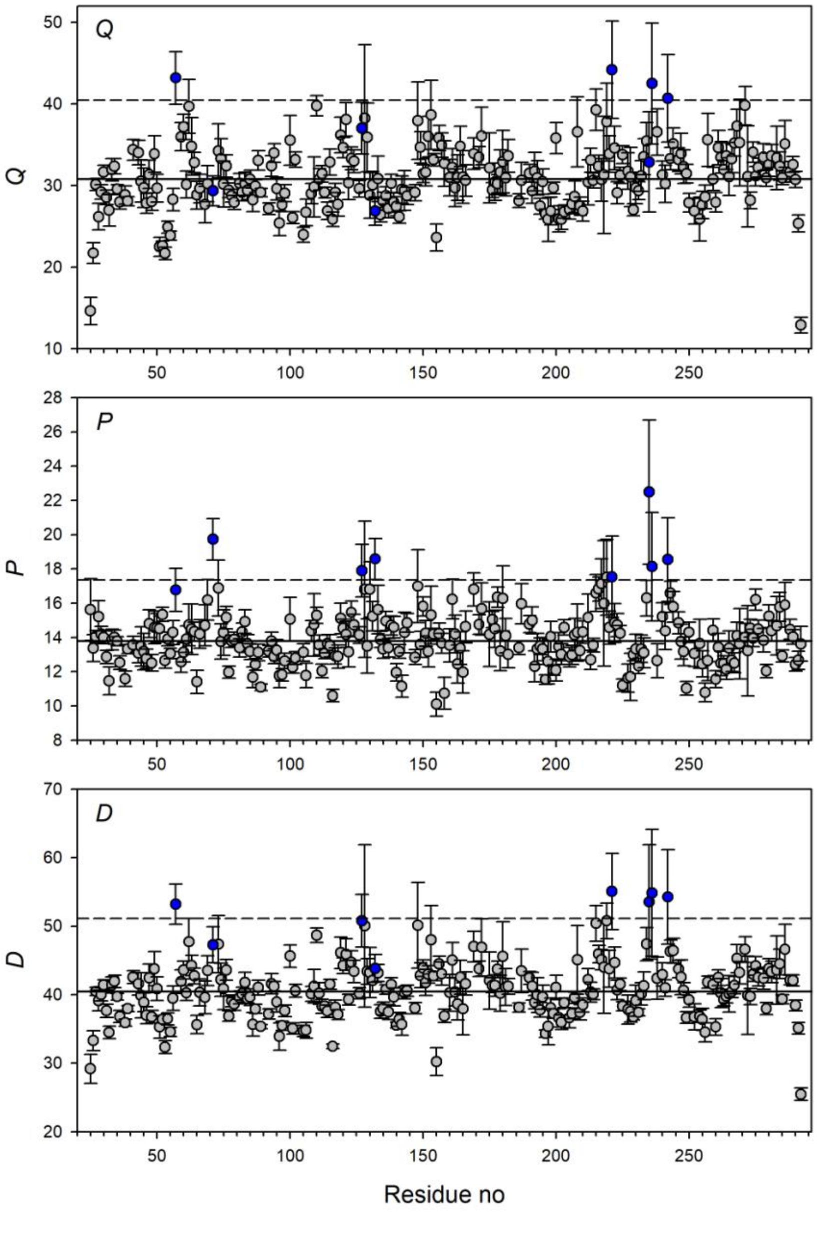

Figure S15

Plots of $Q(\tau_R)$ for GB1 in the rigid molecule approximation (S^2=1.0) at three magnetic field strengths. Color coded vertical lines correspond to the appropriate medians. Intersections of these lines with the $Q(\tau_R)$ curves give evaluations of τ_R at a given magnetic field strength. Dark pink arrow shows τ_R value calculated in the simultaneous MFA analysis of all relaxation data.

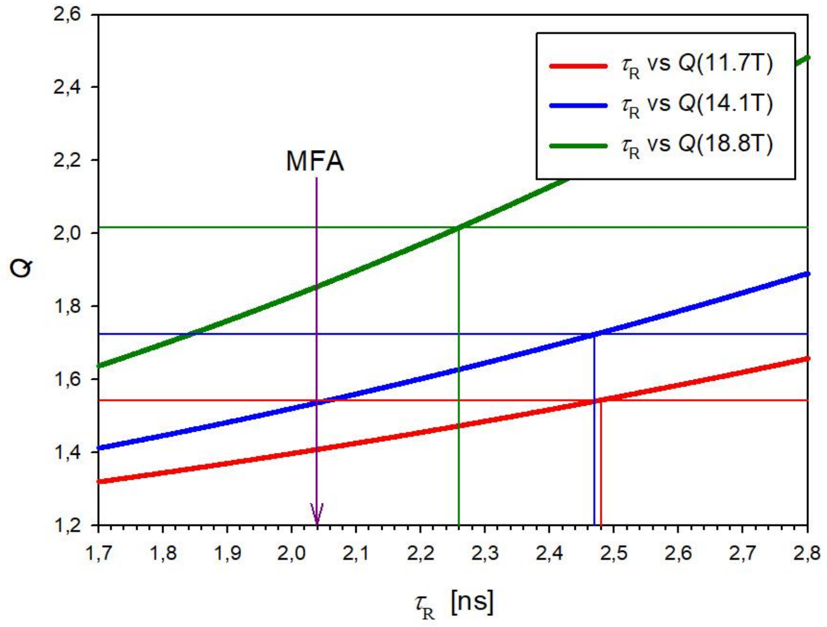

Figure S16

Plots of $Q(\tau_R)$ for ubiquitin in the rigid molecule (S^2=1.0) at two magnetic field strengths. Color coded vertical lines correspond to the appropriate medians. Intersections of these lines with the $Q(\tau_R)$ curves give evaluations of τ_R at a given magnetic field strength. Dark pink arrow shows τ_R value calculated in the simultaneous MFA analysis of all relaxation data.

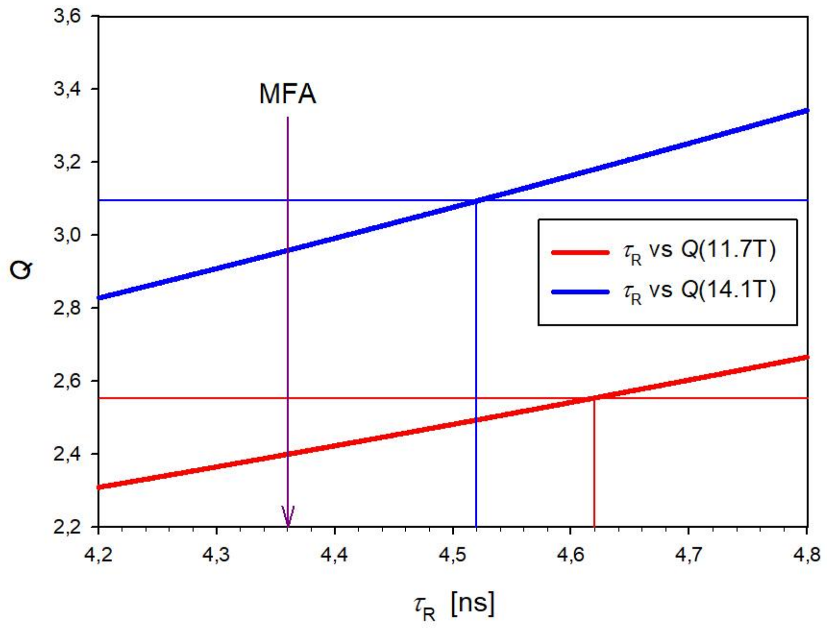

Figure S17

Plots of $Q(\tau_R)$ for S100A1 in the rigid molecule (S^2=1.0) at three magnetic field strengths. Color coded vertical lines correspond to the appropriate medians. Intersections of these lines with the $Q(\tau_R)$ curves give evaluations of τ_R at a given magnetic field strength. Dark pink arrow shows τ_R value calculated in the simultaneous MFA analysis of all relaxation data.

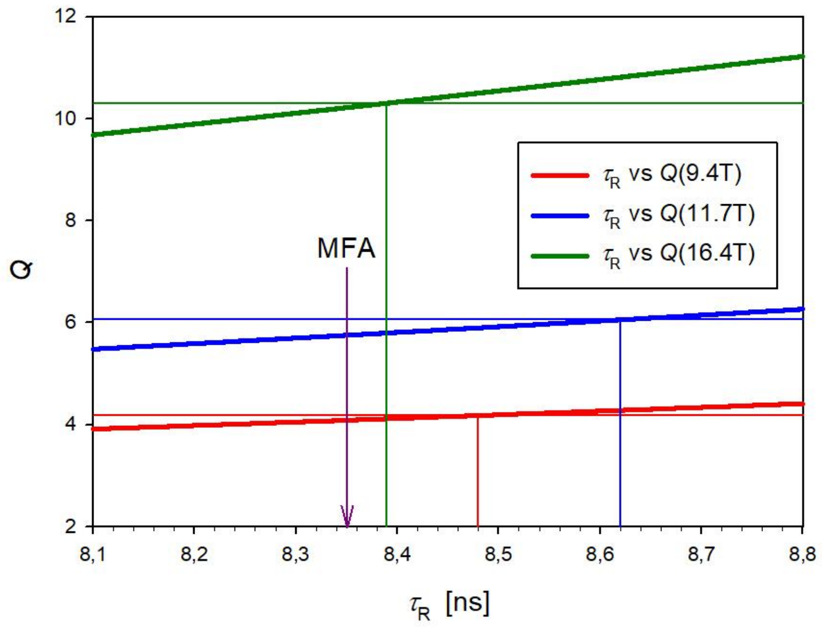

Figure S18

Plots of $Q(\tau_R)$ for PSE4 in the rigid molecule (S^2=1.0) at three magnetic field strengths. Color coded vertical lines correspond to the appropriate medians. Intersections of these lines with the $Q(\tau_R)$ curves give evaluations of τ_R at a given magnetic field strength. Dark pink arrow shows τ_R value calculated in the simultaneous MFA analysis of all relaxation data.

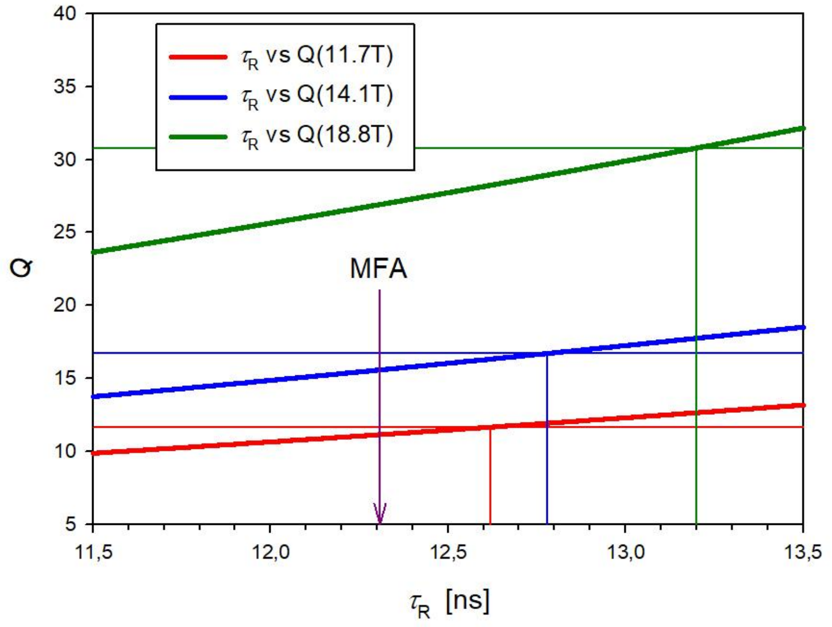

Table S1

As a rule distributions of Q and P values are not gaussian. Ten out of eleven sets of analyzed Q and P values do not pass Shapiro-Wilk test since the calculated W values are smaller than the critical values determined for 5% significance level.

Protein	B_0 [T]	Shapiro-Wilk statistic W		Critical value of W (5% significance level)
		Q	P	
GB1	11.7	**0.974540**	0.957267	0.957540
	14.1	0.920664	0.924558	
	18.8	0.946870	**0.962316**	
UBQ	11.7	0.877437	0.898265	0.959976
	14.1	0.711947	0.787788	
S100A1	9.4	0.920802	0.914296	0.961620
	11.7	0.854964	0.819446	
	16.4	0.855103	0.614072	
PSE4	11.7	0.975360	0.964541	0.987789
	14.1	0.947866	0.978051	
	18.8	0.967148	0.952375	

Table S2

Comparison of the overall correlation times τ_R in ns determined by the model-free approach and from the appropriate $\tilde{Q}$ values. Uncertainties of correlation times are given in parentheses. Those given for the MFA calculations were obtained as standard deviations from 200 Monte Carlo simulations. Unsymmetrical confidence limits of the Q-derived correlation times were calculated from τ values obtained for the first and third Q quartile in the same way as τ_R values were obtained from the median.

Method [a]	GB1 [b]	Ubiquitin [c]	S100A1 [d]	PSE4 [b]
τ_R(MFA)	2.05 (0.02)	4.36 (0.03)	8.35 (0.04)	12.30 (0.08)
$\tau_R(Q)$, 1st B_0	(0.18) 2.48 (0.17)	(0.19) 4.62(0.09)	(0.24) 8.48 (0.19)	(0.45) 12.62 (0.38)
$\tau_R(Q)$, 2nd B_0	(0.05) 2.47 (0.17)	(0.13) 4.52(0.10)	(0.21) 8.62 (0.17)	(0.52) 12.78 (0.54)
$\tau_R(Q)$, 3rd B_0	(0.10) 2.26 (0.12)	---	(0.20) 8.39 (0.26)	(0.50) 13.20 (0.55)

[a] MFA method: all available experimental data were used assuming fully anisotropic tumbling; τ_R averaged according to $\tau_R=0.5/(D_1+D_2+D_3)$. The τ_R values at 1st, 2nd, and 3rd B_0 obtained from Q values

[b] magnetic field strengths: 1st B_0=11.7 T, 2nd B_0=14.1 T, and 3rd B_0=18.8 T

[c] subsequent magnetic field strengths: 1st B_0=11.7 T and 2nd B_0=14.1 T

[d] subsequent magnetic field strengths: 1st B_0=9.4 T, 2nd B_0=11.7 T, 3rd B_0=16.4 T

Table S3

Immunoglobulin-binding domain of streptococcal protein G, GB1. Results of MFA analysis were achieved for ^{15}N relaxation data (R_1, R_2 and *NOE* at 11.7, 14.1, and 18.8 T; 9 relaxation data per residue) of 55 amino acid residues assuming fully anisotropic tumbling. Diffusion constants and their standard deviations (in parentheses) are given in 10^7 rad/s, Euler angles in degrees. Chemical exchange factors Φ and their standard deviations $d\Phi$ are expressed in 10^{18} [s·rad^{-2}].

D_1=9.865 (0.067), D_2=7.286 (0.060), D_3=7.352 (0.064) diffusion constants result

τ_R=2.04 (0.01) ns

θ=–25 (15), ψ=44 (21), φ=–39 (18)

Res. no.	S^2	dS^2	τ_{int} [ps]	$d\tau_{int}$ [ps]	Φ	$d\Phi$
2	0.806	0.015	36.0	10.6	0.12	0.05
3	0.905	0.017	21.0	19.1	0.12	0.06
4	0.914	0.016	0.0	5.5	0.10	0.06
5	0.921	0.015	0.0	7.4	0.22	0.05
6	0.912	0.014	0.0	2.2	0.13	0.06
7	0.897	0.014	0.0	2.4	0.14	0.05
8	0.843	0.017	23.0	14.6	0.18	0.06
9	0.882	0.014	0.0	5.1	0.06	0.05
10	0.866	0.016	63.0	18.0	0.11	0.07
11	0.884	0.018	150.0	42.6	0.16	0.09
12	0.743	0.011	81.0	8.2	0.07	0.05
13	0.795	0.011	67.0	9.8	0.00	0.02
14	0.756	0.013	32.0	7.7	0.13	0.06
15	0.820	0.016	5.0	8.3	0.14	0.06
16	0.907	0.016	0.0	1.8	0.15	0.07
17	0.708	0.035	4381.0	40.9	0.26	0.05
18	0.946	0.017	0.0	0.4	0.11	0.06
19	0.841	0.015	0.0	6.0	0.18	0.06
20	0.912	0.015	0.0	2.5	0.04	0.05
21	0.863	0.020	10.0	11.0	0.15	0.07
22	0.910	0.020	54.0	30.4	0.37	0.08
23	0.909	0.013	0.0	9.3	0.08	0.04
24	0.851	0.017	0.0	8.4	0.23	0.08
25	0.977	0.014	0.0	9.0	0.00	0.00
26	0.955	0.013	0.0	4.5	0.20	0.05
27	0.928	0.016	0.0	6.5	0.07	0.06
28	0.908	0.015	0.0	3.4	0.27	0.06
29	0.901	0.017	1.0	12.0	0.24	0.06
30	0.932	0.013	0.0	17.3	0.14	0.05
31	0.940	0.019	0.0	0.0	0.20	0.08
32	0.909	0.018	0.0	1.4	0.31	0.07
33	0.881	0.015	0.0	2.7	0.15	0.05
34	0.939	0.013	0.0	10.0	0.15	0.05
35	0.883	0.017	0.0	0.0	0.35	0.08
36	0.904	0.014	0.0	15.2	0.11	0.05
37	0.868	0.015	0.0	9.3	0.15	0.05

38	0.838	0.016	4.0	8.8	0.31	0.06
39	0.885	0.016	0.0	3.4	0.23	0.06
40	0.739	0.012	72.0	8.4	0.20	0.06
41	0.566	0.010	40.0	2.9	0.18	0.05
42	0.857	0.015	16.0	13.7	0.24	0.05
43	0.855	0.017	14.0	13.5	0.21	0.07
44	0.923	0.014	119.0	38.0	0.00	0.03
45	0.904	0.014	40.0	22.1	0.10	0.05
46	0.904	0.014	77.0	28.0	0.27	0.05
47	0.919	0.014	0.0	3.4	0.09	0.05
48	0.832	0.017	1.0	7.3	0.17	0.07
49	0.939	0.018	0.0	14.1	0.33	0.06
50	1.000	0.010	0.0	8.9	0.16	0.06
51	0.809	0.039	4398.0	39.2	0.30	0.07
52	0.935	0.015	0.0	9.8	0.15	0.06
53	0.943	0.017	0.0	11.6	0.13	0.08
54	0.930	0.015	0.0	8.2	0.15	0.05
55	0.920	0.015	4.0	18.1	0.19	0.07
56	0.876	0.016	0.0	6.7	0.23	0.08

Table S4

Human ubiquitin. Results of MFA analysis were achieved for ^{15}N relaxation data (R_1 at 9.4 T, R_1, R_2 and *NOE* at 11.7 and 14.1 T, and R_1 and *NOE* at 17.6 T; 9 relaxation data per residue) of 59 amino acid residues assuming fully anisotropic tumbling. Diffusion constants and their standard deviations (in parentheses) are given in 10^7 rad/s, Euler angles in degrees. Chemical exchange factors Φ and their standard deviations $d\Phi$ are expressed in 10^{18} [s·rad^{-2}].

D_1=3.234 (0.030), D_2=3.761 (0.028), D_3=4.469 (0.031) diffusion constants result in τ_R=4.36 (0.02) ns.

θ=−11 (3), ψ=−263 (12), φ=4 (12)

Res. no.	S^2	dS^2	τ_{int} [ps]	$d\tau_{int}$ [ps]	Φ	$d\Phi$
2	0.831	0.004	45.8	2.7	0.37	0.05
3	0.841	0.004	12.7	2.5	0.15	0.05
4	0.926	0.005	18.6	6.8	0.07	0.05
5	0.877	0.005	15.7	3.8	0.11	0.04
6	0.910	0.004	10.4	6.0	0.00	0.01
7	0.877	0.004	35.3	4.2	0.30	0.03
8	0.826	0.004	55.6	3.2	0.13	0.05
9	0.782	0.004	64.6	2.5	0.03	0.05
10	0.787	0.003	67.1	2.4	0.00	0.00
11	0.743	0.002	53.9	1.5	0.20	0.02
12	0.804	0.003	60.1	2.6	0.11	0.03
13	0.877	0.005	41.5	4.9	0.05	0.05
14	0.852	0.004	17.3	3.1	0.20	0.04
15	0.891	0.005	32.5	4.2	0.07	0.05
16	0.795	0.004	36.8	2.0	0.23	0.04
17	0.888	0.004	30.4	4.5	0.43	0.05
18	0.846	0.005	29.5	3.2	0.38	0.05
20	0.866	0.004	33.4	3.2	0.27	0.05
22	0.885	0.005	31.9	4.2	0.06	0.05
23	0.937	0.005	0.0	5.0	0.53	0.06
25	0.925	0.005	15.0	6.4	1.40	0.05
26	0.911	0.004	16.8	4.5	0.12	0.03
27	0.938	0.004	0.0	2.0	0.26	0.05
29	0.911	0.004	40.5	5.2	0.16	0.05
30	0.923	0.004	12.9	5.4	0.13	0.05
32	0.920	0.004	1.1	3.6	0.13	0.04
33	0.873	0.004	21.6	3.2	0.06	0.05
34	0.875	0.005	24.0	3.9	0.12	0.05
35	0.906	0.004	10.2	4.8	0.00	0.02
36	0.771	0.004	1.9	1.4	0.38	0.04
39	0.890	0.003	0.0	1.0	0.12	0.03
40	0.907	0.005	22.0	5.8	0.09	0.04
41	0.876	0.004	12.6	3.8	0.30	0.04
42	0.880	0.005	9.8	4.5	0.12	0.05
43	0.862	0.006	2.7	3.6	0.30	0.05
44	0.892	0.004	17.7	4.9	0.17	0.05
45	0.894	0.005	0.0	3.0	0.24	0.05
46	0.892	0.005	5.9	4.5	0.19	0.05

47	0.854	0.004	46.9	3.1	0.12	0.04
48	0.852	0.003	26.0	2.6	0.18	0.03
49	0.802	0.003	60.8	2.1	0.11	0.02
50	0.881	0.005	22.5	4.6	0.06	0.05
51	0.853	0.006	24.9	3.9	0.10	0.05
52	0.809	0.004	27.5	2.0	0.28	0.04
54	0.864	0.004	7.7	3.1	0.23	0.04
55	0.887	0.005	11.6	4.6	0.21	0.05
56	0.931	0.005	25.8	6.4	0.04	0.05
57	0.898	0.004	1.2	2.5	0.15	0.05
58	0.917	0.004	0.0	2.6	0.17	0.05
59	0.884	0.005	9.3	3.8	0.11	0.04
60	0.877	0.005	25.0	3.5	0.34	0.05
62	0.753	0.004	64.0	2.2	0.06	0.05
63	0.865	0.003	7.3	2.3	0.12	0.03
64	0.915	0.005	21.9	6.5	0.06	0.05
65	0.882	0.004	6.9	3.2	0.16	0.05
66	0.877	0.005	5.0	3.9	0.18	0.05
67	0.902	0.005	24.5	6.2	0.00	0.01
68	0.893	0.005	24.2	5.0	0.15	0.05
70	0.903	0.005	44.5	6.9	0.25	0.05

Table S5

Human A100A1 calcium binding protein in *apo* state. Results of MFA analysis were achieved for ^{15}N relaxation data (R_1, R_2 and *NOE* at 9.4, 11.7 and 16.4 T; 9 relaxation data per residue) of 62 amino acid residues assuming fully anisotropic tumbling. Diffusion constants and their standard deviations (in parentheses) are given as 10^7 rad/s, Euler angles in degrees. Chemical exchange factors Φ and their standard deviations $d\Phi$ are expressed in 10^{18} [s·rad^{-2}].

D_1=2.120 (0.010), D_2=2.095 (0.010), D_3=1.772 (0.011) diffusion constants result in τ_R=8.35 (0.04) ns

Owing to the C_2 symmetry of a dimer molecule, two Euler angles, θ=0 and ψ=0.

φ=22 (4)

Res. no.	S^2	dS^2	τ_{int} [ps]	$d\tau_{int}$ [ps]	Φ	$d\Phi$
3	0.997	0.009	50.5	141.7	0.22	0.82
5	0.936	0.007	72.1	33.3	0.43	1.59
6	0.943	0.007	72.7	30.7	0.21	0.79
7	0.958	0.005	151.4	72.2	0.19	0.70
8	0.955	0.004	40.1	25.4	0.08	0.32
12	0.944	0.006	45.4	24.4	0.22	0.82
15	0.968	0.006	79.6	42.2	0.19	0.70
16	0.947	0.004	91.5	29.0	0.62	2.30
17	0.954	0.003	103.3	26.6	0.46	1.71
20	0.837	0.005	1408.0	72.6	0.73	2.70
22	0.783	0.022	1512.0	224.1	3.61	13.37
23	0.861	0.010	116.3	72.6	1.04	3.86
25	0.885	0.012	998.6	249.1	1.71	6.33
26	0.903	0.008	928.4	130.4	0.64	2.37
27	0.918	0.003	87.5	16.1	0.31	1.15
29	0.895	0.003	143.9	20.7	0.00	0.00
30	0.947	0.006	94.2	37.4	0.11	0.40
31	0.940	0.003	84.9	19.7	0.14	0.52
34	0.946	0.003	62.2	22.3	0.12	0.44
35	0.946	0.003	91.5	22.3	0.03	0.13
36	0.940	0.003	48.1	17.1	0.05	0.17
37	0.953	0.004	84.7	26.0	0.40	1.48
38	0.937	0.004	52.6	18.0	0.13	0.48
39	0.918	0.003	69.9	13.3	0.01	0.03
41	0.921	0.005	49.4	18.7	0.30	1.10
42	0.895	0.003	68.9	13.6	0.11	0.41
43	0.908	0.008	1078.0	165.8	0.24	0.89
45	0.959	0.006	111.8	58.5	0.01	0.05
46	0.921	0.003	147.1	58.7	0.00	0.00
47	0.873	0.003	62.6	6.9	0.00	0.00
48	0.668	0.003	1071.0	18.2	0.00	0.00
51	0.868	0.002	23.9	5.0	0.13	0.48
52	0.932	0.002	99.0	14.3	0.27	1.00
53	0.908	0.003	33.1	6.7	0.21	0.77
54	0.910	0.003	43.9	11.1	0.14	0.52
55	0.928	0.003	48.8	11.6	0.21	0.77

56	0.918	0.002	42.0	9.5	0.24	0.89
58	0.935	0.003	55.1	15.0	0.18	0.66
59	0.934	0.002	95.1	14.6	0.17	0.63
60	0.901	0.002	63.9	7.7	0.34	1.26
61	0.936	0.004	79.7	17.9	0.10	0.37
62	0.872	0.002	46.2	5.9	0.07	0.25
63	0.856	0.002	55.1	6.0	0.28	1.04
64	0.909	0.004	137.6	87.7	0.00	0.00
65	0.894	0.003	122.6	53.2	0.00	0.00
66	0.773	0.006	1944.0	140.3	0.24	0.89
67	0.867	0.015	169.9	218.6	0.00	0.00
68	0.853	0.003	63.5	7.3	0.00	0.00
69	0.886	0.004	57.2	13.3	0.45	1.66
70	0.916	0.006	66.7	15.5	0.10	0.36
71	0.941	0.005	94.3	31.2	0.10	0.36
72	0.892	0.003	39.2	8.8	0.16	0.59
73	0.930	0.003	46.9	14.4	0.11	0.41
74	0.952	0.004	49.6	23.8	0.00	0.01
75	0.966	0.002	33.9	23.9	0.28	1.03
78	0.956	0.003	79.7	23.3	0.00	0.01
79	0.975	0.003	69.8	39.8	0.11	0.41
81	0.959	0.005	56.0	29.4	0.15	0.55
82	0.948	0.004	61.7	23.2	0.00	0.01
84	0.956	0.004	86.4	31.7	0.34	1.25
85	0.907	0.004	46.1	13.7	0.20	0.73
90	0.889	0.003	69.6	12.3	0.00	0.00

Table S6

β-lactamase PSE-4. Results of MFA analysis were achieved for ^{15}N relaxation data (R_1, R_2 and *NOE* at 11.7, 14.1, and 18.8 T; 9 relaxation data per residue) of 223 amino acid residues assuming fully anisotropic tumbling. Diffusion constants and their standard deviations (in parentheses) are given as 10^7 rad/s, Euler angles in degrees. Chemical exchange factors Φ and their standard deviations *d*Φ are expressed in 10^{18} [s·rad^{-2}].

D_1=1.305 (0.009), D_2=1.171 (0.008), D_3=1.587 (0.009)) diffusion constants result in τ_R=12.30 (0.08) ns

θ=–42 (1), ψ=–66 (1), φ=102 (3)

Res. no.	S^2	dS^2	τ_{int} [ps]	$d\tau_{int}$ [ps]	Φ	*d*Φ
25	0.823	0.016	863.0	50.7	0.00	0.23
26	0.922	0.008	93.0	22.0	0.00	0.00
27	0.944	0.008	48.0	11.8	0.42	0.13
28	0.950	0.007	885.0	64.5	0.00	0.09
29	0.928	0.008	35.0	7.3	0.48	0.15
30	0.948	0.011	10.0	8.7	0.66	0.16
31	0.907	0.009	22.0	5.0	0.64	0.15
33	0.944	0.009	0.0	3.8	0.00	0.00
34	0.949	0.011	9.0	9.8	0.94	0.19
35	0.960	0.010	36.0	15.5	0.66	0.17
36	0.940	0.008	11.0	6.7	0.69	0.12
38	0.934	0.010	1.0	5.0	0.16	0.15
39	0.919	0.006	14.0	5.8	0.00	0.02
41	0.947	0.008	27.0	8.4	0.10	0.09
43	0.906	0.009	1.0	3.1	0.75	0.16
44	0.909	0.009	35.0	6.1	1.13	0.22
45	0.906	0.012	22.0	6.6	0.60	0.23
46	0.941	0.016	3.0	9.8	0.52	0.23
47	0.935	0.012	5.0	7.9	0.81	0.20
48	0.935	0.014	7.0	8.4	1.53	0.23
49	0.922	0.014	5.0	7.2	0.65	0.20
50	0.905	0.014	12.0	7.6	1.53	0.28
51	0.927	0.014	29.0	12.6	0.85	0.24
52	0.954	0.009	109.0	39.2	0.00	0.01
53	0.925	0.004	421.0	38.9	0.15	0.09
54	0.940	0.007	83.0	21.0	0.00	0.00
55	0.912	0.006	62.0	7.3	0.00	0.05
56	0.928	0.008	80.0	17.9	0.00	0.01
57	0.912	0.016	21.0	9.4	2.95	0.35
59	0.893	0.007	0.0	1.7	1.11	0.14
60	0.890	0.014	0.0	0.6	1.03	0.23
61	0.986	0.011	22.0	32.7	0.00	0.12
62	0.942	0.013	0.0	3.9	0.94	0.31
63	0.999	0.008	0.0	29.1	0.00	0.13
64	0.958	0.010	0.0	6.1	0.73	0.20
65	0.916	0.010	9.0	5.9	0.11	0.15
66	0.943	0.017	18.0	15.3	0.77	0.29

68	0.994	0.011	218.0	89.5	0.32	0.30
69	0.992	0.015	0.0	24.5	1.27	0.32
71	1.000	0.005	0.0	264.1	1.13	0.24
73	0.996	0.010	72.0	37.4	1.11	0.22
74	0.952	0.013	0.0	4.1	0.68	0.37
75	1.000	0.007	0.0	23.3	0.23	0.24
76	0.941	0.013	0.0	0.1	1.29	0.22
77	0.929	0.010	0.0	3.3	0.10	0.14
78	0.967	0.009	0.0	0.8	0.58	0.18
79	0.973	0.010	0.0	1.9	0.10	0.14
81	0.950	0.013	0.0	3.4	0.43	0.20
82	0.981	0.011	16.0	22.5	0.56	0.18
83	0.966	0.011	0.0	9.9	0.82	0.22
84	0.975	0.008	16.0	16.8	0.05	0.10
85	0.942	0.008	0.0	1.8	0.58	0.18
86	0.884	0.009	10.0	4.1	0.08	0.15
87	0.897	0.008	11.0	4.5	0.81	0.14
88	0.944	0.010	29.0	9.8	0.29	0.18
89	0.864	0.004	30.0	2.4	0.00	0.01
92	0.933	0.006	7.0	5.4	0.03	0.09
93	0.883	0.008	0.0	2.0	1.62	0.17
94	0.910	0.009	9.0	4.3	0.68	0.14
95	0.907	0.011	25.0	7.2	0.85	0.21
96	0.851	0.008	28.0	2.8	0.63	0.15
97	0.873	0.010	34.0	5.3	0.31	0.15
98	0.891	0.010	27.0	5.7	0.86	0.17
100	0.964	0.014	57.0	159.4	0.90	0.22
101	0.902	0.008	41.0	5.5	0.12	0.12
102	0.901	0.008	25.0	4.3	0.80	0.15
105	0.932	0.009	76.0	17.7	0.00	0.00
106	0.908	0.012	22.0	6.9	0.00	0.10
108	0.962	0.011	110.0	50.9	0.47	0.20
109	0.919	0.023	15.0	14.1	1.21	0.38
110	0.964	0.010	6.0	10.9	1.83	0.19
111	0.911	0.010	53.0	8.6	0.72	0.19
112	0.851	0.010	17.0	3.2	1.12	0.17
113	0.861	0.008	35.0	3.4	0.80	0.11
114	0.945	0.013	37.0	17.2	0.70	0.19
115	0.902	0.007	29.0	4.1	1.10	0.14
116	0.813	0.006	29.0	1.8	0.41	0.09
117	0.860	0.013	23.0	4.6	1.16	0.19
118	0.927	0.013	4.0	6.7	0.57	0.17
119	0.925	0.015	2.0	6.3	1.68	0.30
120	0.930	0.012	0.0	1.9	1.37	0.21
121	0.919	0.012	0.0	2.0	1.60	0.29
122	0.969	0.010	0.0	1.2	0.31	0.14
123	0.943	0.011	16.0	8.8	1.63	0.24
124	0.948	0.014	8.0	8.2	1.03	0.26

126	0.987	0.011	0.0	17.2	0.43	0.17
127	1.000	0.008	0.0	123.5	2.26	0.28
128	0.971	0.016	0.0	15.2	4.48	0.50
129	0.971	0.019	0.0	2.4	1.24	0.33
130	1.000	0.006	0.0	13.6	1.22	0.42
131	0.973	0.016	3.0	22.0	1.04	0.28
132	1.000	0.005	0.0	271.9	1.11	0.23
134	0.991	0.014	0.0	5.2	1.60	0.21
135	0.966	0.011	8.0	14.3	0.37	0.17
136	0.953	0.011	0.0	1.7	0.55	0.17
137	0.951	0.012	0.0	3.9	1.13	0.17
138	0.961	0.012	0.0	10.1	0.30	0.19
139	0.958	0.013	5.0	12.4	1.24	0.21
140	0.955	0.014	28.0	17.7	0.80	0.22
141	0.926	0.009	0.0	3.3	0.36	0.12
142	0.940	0.005	28.0	7.3	0.00	0.01
143	0.891	0.012	2.0	3.4	0.22	0.19
144	0.992	0.008	0.0	8.0	0.00	0.08
147	0.969	0.011	0.0	3.1	0.66	0.17
148	0.976	0.009	0.0	2.2	0.11	0.13
149	0.944	0.012	16.0	10.3	1.64	0.38
150	0.979	0.007	150.0	64.7	0.72	0.20
151	0.980	0.010	0.0	15.5	0.75	0.20
152	0.961	0.012	0.0	1.5	0.81	0.23
153	0.987	0.013	0.0	19.0	1.05	0.24
154	0.973	0.013	0.0	1.6	1.69	0.35
155	0.936	0.012	15.0	7.5	0.77	0.17
156	0.947	0.014	0.0	5.8	0.84	0.23
157	0.908	0.011	9.0	5.2	1.33	0.23
158	0.923	0.010	16.0	9.9	0.00	0.05
160	0.930	0.009	7.0	7.3	0.00	0.12
161	0.981	0.013	20.0	30.0	1.54	0.27
162	0.960	0.016	22.0	19.5	0.86	0.30
163	0.904	0.024	0.0	6.5	1.01	0.38
164	0.865	0.020	11.0	6.0	1.11	0.36
165	0.889	0.012	4.0	4.9	0.00	0.18
166	0.956	0.020	0.0	13.8	1.04	0.31
169	0.986	0.013	36.0	56.5	0.80	0.29
171	0.955	0.014	0.0	6.5	1.27	0.22
172	0.913	0.021	9.0	10.0	1.32	0.39
175	0.976	0.012	260.0	82.3	0.34	0.42
176	0.918	0.010	3.0	4.7	1.25	0.18
177	0.954	0.014	39.0	22.8	0.00	0.12
178	0.980	0.013	5.0	20.5	1.15	0.23
179	0.936	0.017	0.0	6.5	0.82	0.34
180	0.927	0.016	0.0	2.0	0.61	0.32
181	0.960	0.015	0.0	6.4	0.48	0.27
182	0.926	0.011	7.0	6.9	0.71	0.19

186	0.961	0.012	0.0	4.7	0.13	0.14
187	0.990	0.008	0.0	8.9	0.87	0.19
190	0.988	0.010	0.0	11.1	0.77	0.19
191	0.945	0.015	10.0	10.2	1.17	0.34
192	0.963	0.021	0.0	2.8	0.26	0.23
193	0.927	0.010	1.0	4.2	0.39	0.20
194	0.959	0.010	19.0	14.8	0.09	0.14
195	0.900	0.015	1.0	4.2	0.99	0.29
196	0.859	0.006	23.0	3.6	0.00	0.04
197	0.835	0.027	27.0	10.4	0.96	0.44
198	0.920	0.010	92.0	31.3	0.00	0.00
199	0.913	0.006	15.0	4.9	0.00	0.00
200	0.872	0.012	13.0	5.0	1.04	0.23
201	0.938	0.007	19.0	9.7	0.00	0.03
202	0.947	0.006	41.0	10.1	0.00	0.04
203	0.952	0.013	0.0	6.6	0.63	0.18
205	0.950	0.008	18.0	10.6	0.00	0.10
206	0.944	0.009	5.0	7.6	0.62	0.15
207	0.960	0.012	29.0	18.4	0.80	0.17
208	0.978	0.012	0.0	1.8	0.53	0.22
209	0.968	0.006	17.0	14.1	0.00	0.04
210	0.963	0.010	3.0	10.7	0.49	0.17
212	1.000	0.003	0.0	11.0	1.10	0.19
213	0.904	0.016	0.0	0.0	1.57	0.24
214	0.904	0.011	14.0	5.7	0.66	0.18
215	0.950	0.016	18.0	18.0	1.25	0.37
216	0.940	0.013	33.0	15.6	1.68	0.21
217	0.963	0.012	75.0	62.3	1.09	0.20
218	1.000	0.019	0.0	196.5	1.07	0.65
219	1.000	0.014	0.0	10.3	0.22	0.25
220	0.922	0.013	5.0	6.4	1.75	0.26
221	0.905	0.026	0.0	8.6	3.85	0.52
222	0.984	0.017	0.0	13.0	0.70	0.35
223	0.962	0.013	0.0	12.4	0.53	0.28
224	0.928	0.014	0.0	4.0	1.00	0.21
225	0.886	0.008	3.0	3.1	0.31	0.14
227	0.895	0.006	13.0	3.5	0.00	0.09
228	0.860	0.020	25.0	6.0	0.18	0.24
229	0.933	0.008	29.0	7.2	0.00	0.07
230	0.879	0.013	16.0	6.0	1.53	0.26
231	0.915	0.011	2.0	4.2	0.29	0.14
232	0.902	0.017	0.0	3.5	1.03	0.31
233	0.907	0.015	3.0	5.0	1.59	0.27
234	0.915	0.021	21.0	11.6	1.89	0.40
235	1.000	0.013	0.0	31.8	4.23	0.70
236	0.934	0.022	0.0	7.2	3.18	0.46
238	0.885	0.013	24.0	5.6	1.06	0.29
240	0.974	0.015	0.0	8.1	0.93	0.27

241	1.000	0.007	0.0	270.1	0.16	0.17
242	0.989	0.008	398.0	105.6	1.22	0.30
243	0.979	0.012	0.0	3.9	1.12	0.24
244	0.941	0.016	7.0	12.6	2.21	0.31
246	0.944	0.014	0.0	6.9	1.41	0.25
247	0.965	0.019	0.0	10.3	0.79	0.38
248	0.921	0.011	4.0	6.2	0.65	0.20
249	0.884	0.011	0.0	0.7	0.86	0.17
250	0.974	0.013	0.0	10.9	0.59	0.21
252	0.948	0.011	4.0	8.6	0.21	0.18
254	0.934	0.017	94.0	46.0	0.43	0.21
255	0.829	0.022	39.0	8.6	0.63	0.32
256	0.815	0.005	32.0	2.4	0.00	0.03
257	0.923	0.010	33.0	8.5	0.67	0.18
259	0.927	0.014	0.0	6.6	1.13	0.23
260	0.894	0.013	0.0	1.9	0.53	0.23
261	0.904	0.016	5.0	5.8	0.88	0.24
262	0.925	0.012	0.0	4.3	0.80	0.28
263	0.928	0.012	0.0	5.2	0.41	0.20
264	0.904	0.015	0.0	2.0	0.52	0.22
265	0.901	0.016	22.0	8.6	1.65	0.33
266	0.895	0.010	17.0	5.3	0.79	0.18
269	0.913	0.017	13.0	8.6	0.58	0.26
271	0.881	0.013	14.0	4.4	1.48	0.24
272	0.950	0.021	33.0	30.9	0.50	0.32
273	0.909	0.016	16.0	7.8	1.33	0.29
274	0.914	0.036	32.0	25.0	0.50	0.60
275	0.955	0.011	30.0	16.5	0.38	0.19
276	0.919	0.014	9.0	6.8	1.21	0.23
278	0.976	0.008	129.0	54.1	0.81	0.22
279	0.919	0.012	9.0	6.7	1.52	0.22
280	0.960	0.010	0.0	6.5	0.72	0.19
281	0.927	0.010	2.0	5.6	0.16	0.15
283	0.944	0.013	3.0	7.5	1.24	0.23
284	0.955	0.011	17.0	13.6	1.19	0.23
285	0.966	0.013	0.0	6.9	0.50	0.27
286	0.971	0.009	2.0	13.4	0.69	0.18
288	0.934	0.009	17.0	6.6	0.60	0.14
289	0.941	0.016	4.0	8.7	1.55	0.33
290	0.997	0.003	380.0	64.8	0.71	0.08
291	0.945	0.012	25.0	12.5	0.73	0.19
292	0.914	0.014	12.0	7.2	0.38	0.19
